# Hyperglycemia Induces Trained Immunity in Macrophages and Their Precursors and Promotes Atherosclerosis

**DOI:** 10.1161/CIRCULATIONAHA.120.046464

**Published:** 2021-07-13

**Authors:** Laurienne Edgar, Naveed Akbar, Adam T. Braithwaite, Thomas Krausgruber, Héctor Gallart-Ayala, Jade Bailey, Alastair L. Corbin, Tariq E. Khoyratty, Joshua T. Chai, Mohammad Alkhalil, André F. Rendeiro, Klemen Ziberna, Ritu Arya, Thomas J. Cahill, Christoph Bock, Jurga Laurencikiene, Mark J. Crabtree, Madeleine E. Lemieux, Niels P. Riksen, Mihai G. Netea, Craig E. Wheelock, Keith M. Channon, Mikael Rydén, Irina A. Udalova, Ricardo Carnicer, Robin P. Choudhury

**Affiliations:** 1 Division of Cardiovascular Medicine, Radcliffe Department of Medicine, University of Oxford, UK (L.E., N.A., A.T.B., J.B., J.T.C., M.A., K.Z., R.A., T.J.C., M.J.C., K.M.C., R.C., R.P.C.).; 2 CeMM Research Center for Molecular Medicine of the Austrian Academy of Sciences, Vienna, Austria (T.K., A.F.R., C.B.).; 3 Division of Physiological Chemistry II, Department of Medical Biochemistry and Biophysics, Karolinska Institutet, Stockholm, Sweden (H.G.-A., C.E.W.).; 4 Department of Respiratory Medicine and Allergy (H.G.-A., C.E.W.), Karolinska University Hospital, Stockholm, Sweden.; 5 Department of Medicine (H7) (J.L., M.R.), Karolinska University Hospital, Stockholm, Sweden.; 6 The Kennedy Institute of Rheumatology, University of Oxford, UK (A.L.C., T.E.K., I.A.U.).; 7 Institute of Artificial Intelligence and Decision Support, Center for Medical Statistics, Informatics, and Intelligent Systems, Medical University of Vienna, Austria (C.B.).; 8 Bioinfo, Plantagenet, ON, Canada (M.E.L.).; 9 Department of Internal Medicine, Radboud University Medical Centre, Nijmegen, The Netherlands (N.P.R.., M.G.N.).; 10 Department for Genomics & Immunoregulation, Life and Medical Sciences Institute (LIMES), University of Bonn, Germany (M.G.N.).

**Keywords:** diabetes mellitus, epigenetics, glucose, inflammation, macrophages

## Abstract

**Background:**

Cardiovascular risk in diabetes remains elevated despite glucose-lowering therapies. We hypothesized that hyperglycemia induces trained immunity in macrophages, promoting persistent proatherogenic characteristics.

**Methods:**

Bone marrow–derived macrophages from control mice and mice with diabetes were grown in physiological glucose (5 mmol/L) and subjected to RNA sequencing (n=6), assay for transposase accessible chromatin sequencing (n=6), and chromatin immunoprecipitation sequencing (n=6) for determination of hyperglycemia-induced trained immunity. Bone marrow transplantation from mice with (n=9) or without (n=6) diabetes into (normoglycemic) *Ldlr* ^−/^^−^ mice was used to assess its functional significance in vivo. Evidence of hyperglycemia-induced trained immunity was sought in human peripheral blood mononuclear cells from patients with diabetes (n=8) compared with control subjects (n=16) and in human atherosclerotic plaque macrophages excised by laser capture microdissection.

**Results:**

In macrophages, high extracellular glucose promoted proinflammatory gene expression and proatherogenic functional characteristics through glycolysis-dependent mechanisms. Bone marrow–derived macrophages from diabetic mice retained these characteristics, even when cultured in physiological glucose, indicating hyperglycemia-induced trained immunity. Bone marrow transplantation from diabetic mice into (normoglycemic) *Ldlr* ^−/^^−^ mice increased aortic root atherosclerosis, confirming a disease-relevant and persistent form of trained innate immunity. Integrated assay for transposase accessible chromatin, chromatin immunoprecipitation, and RNA sequencing analyses of hematopoietic stem cells and bone marrow–derived macrophages revealed a proinflammatory priming effect in diabetes. The pattern of open chromatin implicated transcription factor Runt-related transcription factor 1 (Runx1). Similarly, transcriptomes of atherosclerotic plaque macrophages and peripheral leukocytes in patients with type 2 diabetes were enriched for Runx1 targets, consistent with a potential role in human disease. Pharmacological inhibition of Runx1 in vitro inhibited the trained phenotype.

**Conclusions:**

Hyperglycemia-induced trained immunity may explain why targeting elevated glucose is ineffective in reducing macrovascular risk in diabetes and suggests new targets for disease prevention and therapy.

## Abstract 2

Supplemental Digital Content is available in the text.

**Clinical Perspective**

**What Is New?**

- Hyperglycemia induces a trained immunity in bone marrow progenitor cells by inducing persistent epigenetic modifications.
- Hyperglycemia-induced trained immunity persists after differentiation into macrophages.
- Hematopoietic stem cells transplanted from mice with diabetes to euglycemic mice promote exaggerated atherosclerosis.

**What Are the Clinical Implications?**

- Hyperglycemia induces a trained immunity phenotype that can be identified in circulating leukocytes from patients with type 2 diabetes.
- Hyperglycemia-induced trained immunity may explain why conventional treatments that focus on glucose lowering are ineffective at reducing atherosclerotic vascular disease risk in diabetes.

Diabetes is associated with a high risk of atherosclerosis and its complications, including myocardial infarction.^1^ Hyperglycemia is a cardinal feature in both type 1 and type 2 diabetes, and treatments have focused largely on lowering blood glucose. Intensive glucose-lowering therapy is effective in lowering vascular risk in patients with type 1 diabetes, but the effect is deferred for several years^2^ and temporally disconnected from the control of blood glucose.^3^ In type 2 diabetes, glucose reduction has shown either no effect or a modest effect on atherosclerosis-related vascular outcomes such as acute myocardial infarction.^4–6^ Persistent risk of cardiovascular complications, even after glucose lowering, has been called the legacy effect or metabolic memory,^4,7^ but the underlying mechanisms remain obscure.

Atherosclerosis is a chronic inflammatory disease characterized by the deposition and retention of modified lipoproteins and the accumulation of immune cells in the walls of large arteries. Macrophages are key in all stages of atherosclerosis and are widely regarded as therapeutic targets.^8^ They are heterogeneous and functionally responsive to microenvironmental cues. Macrophages have been subtyped into opposing phenotypes, namely proinflammatory (M1), in response to the Toll-like receptor ligand lipopolysaccharide (LPS) and interferon-γ (IFN-γ), or tissue reparative (M2), for example, in response to interleukin (IL)-4, although in vivo their functional scope is undoubtedly more complex.^9^

Hyperglycemia exacerbates atherosclerosis progression and retards plaque regression,^10^ with increased expression of proinflammatory genes and resistance to induction of M2-associated gene expression.^10,11^ In monocytes and macrophages, cellular energetic pathways are governed by an interplay between cell-intrinsic and environmental stimuli, which, in turn, markedly affect immune-related function and gene expression.^12^ In response to IL-4, M2 macrophages primarily metabolize fatty acids and rely on oxidative phosphorylation.^13^ In contrast, M1 macrophages require glucose and a shift to aerobic glycolysis, similar to the Warburg effect in cancer.^14,15^ These metabolic changes occur as a consequence of cytokine stimulation but can also themselves determine macrophage function.^16^ Furthermore, recent reports indicate that changes in monocyte and macrophage metabolism, including aerobic glycolysis or activation of the mevalonate pathway,^17,18^ induce long-term innate immune cell memory (called trained immunity) through epigenetic modifications and chromatin structure alterations.^19^ The long-term persistence of circulating trained monocytes is driven by reprogramming of their bone marrow (BM) progenitors.^20^

We hypothesized that trained immunity in response to elevated glucose accounts for diabetic hyperglycemic memory in relation to atherosclerosis. Accordingly, we sought to determine whether hyperglycemia induced disease-relevant changes in monocyte and macrophage function and whether these changes persisted after restoration of normal glucose, implying fundamental reprogramming. We combined studies of cellular function, metabolomics, transcriptomics, and epigenomics to define how hyperglycemia alters metabolism to modulate long-term activation through epigenetic modifications. We explored how these changes may occur at the hematopoietic stem cell (HSC) level and in differentiated macrophages. Functional significance is tested through BM transplantation in a mouse model of atherosclerosis, and the findings are validated by interrogation of macrophages extracted from human peripheral blood and from atherosclerotic plaques by laser capture microdissection (LCM). Our findings may explain the resistance of macrovascular complications of diabetes to conventional glucose-lowering treatments.

## Methods

### Data Resources

The data generated in this study have been deposited in the National Center for Biotechnology Information Gene Expression Omnibus and are accessible through accession number GSE176068.

### Animals

Murine BM transplantation experiments was performed at The Jackson Laboratory (Bar Harbor, ME) in vivo facility in accordance with the Institutional Animal Care and Use Committee regulations (Association for Assessment and Accreditation of Laboratory Animal Care accredited facility, project reference 70195 IVAT). All other animal protocols were conducted in accordance with the UK Home Office under the guidance of the operation of the Animals (Scientific Procedures) Act 1986, complying to all ethical regulations and the institutional review board guidelines (project license 30/3374; January 27, 2016). Wild-type C57BL/6J (12–14 weeks old) were randomly assigned to control or diabetic experimental groups. Diabetes was induced through an intraperitoneal injection of streptozotocin at a low dose range (42–45 mg·kg^−1^·/d^−1^) over 5 consecutive days. After 6 weeks after the final streptozotocin injection, diabetic mice with nonfasted blood glucose levels >13.9 mmol/L were euthanized, and tissue was collected.

### Cell Lines

All cell lines were grown at 37 °C and 5% CO_2_. L929 cells were grown in Roswell Park Memorial Institute medium supplemented with 10% fetal bovine serum (FBS), 2% penicillin-streptomycin, and 2 mmol/L glutamine. L929 cells were grown to full confluence for 7 to 8 days, and the conditioned media was removed, divided into aliquots, and stored at −20 °C until required. Skin-derived mouse endothelial cells were cultured in 25 mmol/L glucose DMEM with 10% FBS, 2% penicillin-streptomycin, and 2 mmol/L glutamine.

### BM-Derived Macrophages

BM-derived macrophages (BMDMs) used in this study were generated by flushing BM from murine femurs and tibias and plating on non–tissue culture–treated petri dishes in complete 5 mmol/L glucose DMEM (supplemented with 10% FBS, 2% penicillin-streptomycin, and 2 mmol/L glutamine) plus 20% L929 cell–conditioned media for differentiation. After differentiation for 7 days, BMDMs were positively selected, plated at a density of 2×10^5^ cells/mL, and stimulated in complete 5 mmol/L glucose DMEM (unless stated otherwise) with either 10 ng/mL recombinant murine interferon-γ (IFN-γ; R&D Systems, UK) plus 100 ng/mL LPS (Sigma-Aldrich, UK), 10 ng/mL recombinant mouse IL-4 (R&D Systems, UK), or media-only control for 16 hours before use. BMDMs were also simultaneously cultured in various glycemic conditions (5 mmol/L glucose DMEM with the addition of glucose to make a final concentration of either 11 or 20 mmol/L glucose) or an osmotic control condition (mannitol; 5 mmol/L glucose DMEM plus 15 mmol/L mannitol). Metabolic studies were performed in the absence or presence of 10 mmol/L dichloroacetate (Sigma-Aldrich, UK) or 1 mmol/L 2-deoxy-d-glucose (Sigma-Aldrich, UK).

### HSC Isolation

HSCs were isolated with the MagniSort Mouse Hematopoietic Lineage Depletion Kit (ThermoFisher Scientific, UK) by flushing BM from murine femurs and tibias and incubating with markers to remove lineage committed cells (CD3, CD4, B220, Ter119, and Gr1) and enrich for HSCs. HSCs were cultured in 5 mL fluorescence-activated cell sorting tubes in complete 5 mmol/L DMEM (or 20 mmol/L glucose or mannitol for indicated wild-type stimulations) with the addition of 100 ng/mL LPS plus 10 ng/mL IFN-γ or 10 ng/mL recombinant mouse IL-1β (R&D Systems, UK) for 16 hours for Western blot analysis or 2 hours for assay for transposase accessible chromatin sequencing (ATAC-seq) analysis.

### BMDM In Vitro Memory Model

Mouse BM was differentiated into BMDMs as described above in 5 or 20 mmol/L glucose for 1, 2, 4, or 7 days. BMDMs were positively selected through adhesion, replated into complete 5 mmol/L DMEM, and left to incubate for 48 hours to allow a period of glucose normalization. BMDMs were then stimulated for use with 10 ng/mL IFN-γ plus 100 ng/mL LPS or control media for 16 hours in the absence or presence of 0.25, 0.5, 1, 5, or 10 µmol/L Ro5-3335 (Merck Millipore, UK) before use for gene expression analysis.

### Mouse Monocyte Isolation

Murine monocytes were isolated from peripheral blood with the EasySep Mouse Monocyte Isolation Kit (Stemcell Technologies, Canada) according to the manufacturer’s protocol. Cells were used immediately for static adhesion assay.

### Seahorse XF^e^96 Real-Time ATP Rate Assay Kit

Extracellular flux analysis was performed with the XFp Real-Time ATP Rate Assay kit on BMDMs (8×10^4^ cells per well; 5 replicate wells per animal and per condition), according to the manufacturer’s instructions. Three baseline oxygen consumption rate and extracellular acidification rate measurements were taken before varying concentrations of glucose (5 or 20 mmol/L) or control (mannitol; injection port A) assay medium were injected. After a further 7 measurements, compounds oligomycin (2 µmol/L; injection port B) and combined antimycin A (0.5 µmol/L) and rotenone (0.5 µmol/L; injection port C), from the XFp Real-Time ATP Rate Assay kit, were sequentially injected with 3 subsequent measurements. The amount of ATP produced by glycolysis and the ATP rate index (mitoATP production rate/glycolytic ATP production rate) were automatically calculated with Wave (version 2.6, Agilent).

### Static Adhesion Assay

Skin-derived mouse endothelial cells were grown to confluence on glass coverslips in 12-well plates and activated by 10 ng/mL recombinant mouse tumor necrosis factor-α (R&D Systems, UK) for 16 hours. Unstimulated or IFN-γ+LPS–stimulated peripheral blood monocytes (or BMDMs from streptozotocin diabetic mice cultured in 5 mmol/L glucose) were fluorescently labeled green with the PKH67 fluorescent cell linker kit (Sigma-Aldrich, UK) according to the manufacturer’s instructions. Labeled cells were added to endothelial cells at a density of 5×10^4^ cells per coverslip for 1 hour before media and unattached cells were removed, washed with phosphate buffered saline (PBS), and fixed in 4% paraformaldehyde. Four images per coverslip were acquired; the number of adhered, labeled cells was counted; and the images per coverslip were averaged. Displayed images have been equally brightness adjusted on ImageJ.

### Foam Cell Assay

Unstimulated or stimulated (LPS+IFN-γ) BMDMs were grown on glass coverslips in 12-well plates and incubated with 25 µg/mL DiI-acetylated low-density lipoprotein (LDL; Bioquote, US) for 48 hours. Media and remaining DiI-acetylated LDL were removed, and cells were washed 3 times with PBS and fixed in 4% paraformaldehyde. Lipid was stained with Oil Red O, and coverslips were DAPI mounted. Phase-contrast images of the Oil Red O staining and fluorescent images of the nuclei were taken (4 images per coverslip) and used to quantify the amount of lipid or number of cells, respectively, with ImageJ software. Lipid area per image was then normalized to the number of cells per image, and results are expressed as fold change over 5 mmol/L glucose control samples (for BMDM glucose experiments) and control samples (for diabetic BMDM experiments). Displayed images have been uniformly brightness adjusted.

### Diabetic BM Transplantation Experiment

BM transplantation experiments were performed at The Jackson Laboratory. Diabetes was induced in CD68–green fluorescent protein (GFP) transgenic mice as previously described for 4 weeks. Diabetic CD68-GFP mice with nonfasted blood glucose levels >13.9 mmol/L and control CD68-GFP mice of the same age were used as BM donors. BM was collected from donor mice, and 5×10^6^ viable cells were intravenously injected into lethally irradiated (2×500 rads) *LdLr*^−/^^−^ recipient B6.129S7-Ldlr-tm1Her/J mice (JAX stock No. 002207). After engraftment, all recipient animals were placed on a Western diet (Open Source Diets D12079B;1% fat, 40% kcal) for 12 weeks. Whole blood was collected at 4, 8, and 12 weeks after engraftment for flow cytometric analysis of peripheral blood mononuclear cells (PBMCs) and to monitor engraftment levels and assess leukocyte population numbers. Engraftment, assessed every 4 weeks after engraftment, was monitored by staining cells with anti-CD45 and assessing the relative proportion of CD45+ GFP+ cells among total CD45+ population. At 12 weeks, mice were euthanized, whole blood was collected, and sera were processed for triglycerides, nonesterified fatty acids, LDL, and high-density lipoprotein cholesterol measurements. Mice were perfused with PBS, followed by perfusion and fixation with 4% paraformaldehyde overnight. The heart and aorta were dissected and placed in 70% ethanol until processing.

### Aortic Root Plaque Analysis and Immunofluorescence

To quantify plaque burden, paraformaldehyde-fixed aortic roots were placed in optimal cutting medium and sectioned on a plane parallel to the atria. To quantify plaque volume, plaque lipid content,^21^ and collagen content, 5 sections, evenly distributed through the aortic root, were Masson-Goldner trichrome stained (Merck, UK). Briefly, sections were refixed in Bouin solution at room temperature for 1 hour, washed in running water for 5 minutes, and then put through the Masson-Goldner staining kit protocol from the second 70% ethanol incubation according to the manufacturer’s protocol timings. Xylene-wet slides were then mounted with Neo-Mount and sealed with cover glass. Plaque characteristics were quantified on Image Pro Plus software version 6.0 (Media Cybernetics, Silverspring, MD), and data were displayed as an average of all sections.

Immunofluorescence was then performed on 6 adjacent sections. Sections were stained for macrophage content with GFP (rabbit anti-GFP, A6455, Invitrogen) or the macrophage marker galectin-3 (goat anti–galectin 3, AF1197, R&D Systems), for smooth muscle cell content with α-actin (rabbit anti–smooth muscle actin, ab32575, Abcam PLC), and for the epigenetic marks H3K4me3 (rabbit polyclonal to histone H3 [trimethyl K4], Ab8580, Abcam PLC) and H3K27ac (rabbit polyclonal to histone H3 [acetyl K27], Ab4729, Abcam PLC). For immunofluorescence staining, sections were rehydrated with PBS for 5 minutes at room temperature and blocked for 1 hour with DAKO blocking buffer (Agilent, UK). Primary antibody was diluted to 1 µg/mL in DAKO and added to sections for overnight at 4 °C. Secondary antibodies were diluted 1:200 in DAKO and added for 1 hour at room temperature (donkey anti-rabbit [A488], A21206, Life Technologies; donkey anti-goat [A594], A11058, ThermoFisher Scientific). Sections were finally DAPI mounted (glycerol mounting medium with DAPI and DABCO, Abcam, UK). Fluorescent images were analyzed with Image J software.

### Gene Expression

Total RNA was isolated with RNeasy Plus Mini kit (Qiagen, UK) and quantified with a Nanodrop 2000 UV-visible spectrophotometer. cDNA synthesis was performed with 0.2 to 2 µg RNA in a polymerase chain reaction (PCR) with QuantiTect reverse transcription kit (Qiagen, UK), according to manufacturer’s instructions. Real-time quantitative PCR was performed on 5 ng/μL cDNA with Taqman probes specific for *B2m* (Mm00437762), *Il-6* (Mm00446190), *Il-1β* (Mm00462531_m1), *iNos* (Mm00440502), *Ym1* (Mm00657009), and *Fizz1* (Mm00556208). Quantitative PCR was performed with Taqman universal master mix II with UNG on QuantStudioTM 7 Flex Real-Time PCR System (ThermoFisher). All PCR data were normalized to internal housekeeper gene expression (*B2*m) and analyzed by the 2^−ΔCT^ method to calculate relative mRNA expression.

### Western Blot Analysis

Cells were washed in ice-cold PBS and lysed in radioimmunoprecipitation assay lysis buffer (150 mmol/L NaCl, 1.0% IGEPAL CA-630, 0.5% sodium deoxycholate, 0.1% SDS, 50 mmol/L Tris, pH 8.0) supplemented with protease and phosphatase inhibitors. Protein concentration was determined by bicinchoninic acid assay (Pierce BCA Protein Assay Kit, ThermoFisher Scientific, UK) according to assay instructions. Equal concentrations of protein were loaded and run on an SDS-PAGE gel (4% to 12% Bis-Tris Gel, Life Technologies, UK) to separate. Proteins were transferred onto a nitrocellulose membrane (Bio-Rad, UK) and probed with specific antibodies as indicated with β-actin (mouse anti-β-actin [15G5A11/E2], Invitrogen) as a housekeeper and loading control. Membranes were developed (SuperSignal West Dura Extended Duration Substrate, Thermo Scientific, UK) and detected by chemiluminescence on a Biorad Image Station.

### Human Samples and LCM Isolation of Plaque Macrophages

All clinical investigations were conducted in accordance with the Declaration of Helsinki, and samples were stored under the UK Human Tissue Act (2004). The study was approved by the relevant ethics review board, and all subjects provided written informed consent. Patients awaiting carotid endarterectomy were recruited, and carotid plaques were collected freshly at time of surgery (patient characteristics summarized in Excel File I in the Data Supplement). Explanted carotid plaques were washed in ice-cold sterile PBS, snap-frozen in optimal cutting medium, and stored at −80 °C until processed.

LCM was carried out with a PALM Microbeam LCM system (Carl Zeiss GmbH, Germany) using an adapted “guide slide” approach.^22,23^ Carotid plaque samples were freshly sectioned at 15 µm. Guide slides consisted of 3 consecutive sections: The first section was stained with the Masson-Goldner staining kit protocol to aid in determination of microanatomic locations, and the next 2 were immunostained for either macrophages (mouse antihuman CD68 [clone KP-1], Dako, Cambridge UK) or smooth muscle cells (mouse antihuman α-actin [clone 1A4], Dako, Cambridge UK). The subsequent sections (up to 15) were cut onto LCM membrane slides (ThermoFisher Scientific, UK) and cresyl violet stained ready for immediate processing. Macrophage cell clusters were identified manually on the basis of the guide-slide staining using PALM RoboSoftware (version 4.5, Carl Zeiss GmbH, Germany). A total combined area of interest between 3 and 5×10^6^ µm^2^ were laser-captured on a Zeiss PALM AdhesiveCap (Carl Zeiss GmbH, Germany). Cells were immediately lysed, snap-frozen on dry ice, and stored at −80 °C until the RNA was extracted with the RNEasy micro kit (Qiagen, UK), according to the manufacturer’s protocol.

### Human Plaque Macrophage Microarray

Isolated plaque macrophage RNA concentration was determined with the Agilent RNA 6000 Pico LabChip on the Agilent Bioanalyzer 2100 (Agilent Technologies, US). Samples with an RNA integrity number >5 were taken forward to RNA amplification, biotinylation, and gene expression microarray analysis. These samples were submitted to Cambridge Genomic Services (Department of Pathology, University of Cambridge, UK) for further processing. Amplification and biotinylation were performed with the Ovation Pico WTA V2 kit (Nugen Technologies Inc, US), and then hybridization was varied out in random order on 2 Illumina Human HT12 v4.0 BeadChips (Illumina, US) to minimize batch/chip variation. Illumina BeadArrays were processed with the Bioconductor beadarray package (version 2.24.0)^24,25^ using normal exponential normalization. Probe sets were collapsed per gene on the basis of the maximum interquartile range to avoid extreme values but capture variability across all samples. Unannotated and control probe sets were excluded from further analysis. Differential expression (DE) of the final list of 21 036 genes was assessed with the Bioconductor limma package (version 3.30.13)^26^ to compare diabetic with control, adjusting for symptomatic/asymptomatic status. Given the availability of orthogonal data sets and 8 samples per group, we used a relatively lenient adjusted value of *P*<0.2 with no fold change cutoff to select genes to pursue.

### Human Monocyte Isolation

Peripheral venous blood (15–20 mL) was obtained from healthy donors in K2-EDTA–coated tubes (BD Vacutainer, New Jersey) and processed within 1 hour. Plasma and a mononuclear cell–enriched fraction were obtained by centrifuging whole blood on Accuspin Histopaque-1077 columns (Sigma-Aldrich, US) according to the manufacturer’s instructions. The mononuclear cell–enriched fraction was taken forward for monocyte isolation with a negative selection bead isolation kit (EasySep human monocyte enrichment kit without CD16 depletion, StemCell Technologies, Canada), following the manufacturer’s described protocol.

### Human Peripheral Blood Mononuclear Cell Isolation and Stimulation

Patients with diabetes and control patients were recruited to clinical studies after local and regional ethical approval at the Karolinska Institute, Stockholm (2011/1002-31/1 and 2009/1881-31/1). PBMCs were isolated from peripheral blood samples collected after overnight fasting with SepMate (StemCell Technologies) and Lymphoprep (StemCell Technologies) according to manufacturer’s instructions. Isolated PBMCs were frozen and stored in 90% FBS and 10% dimethyl sulfoxide.

Stored PBMCs were thawed and washed in PBS by centrifugation (500*g* for 10 minutes), viable cells were counted and plated into individual wells of a 12-well tissue culture plate in physiological (low) glucose DMEM, 10% FBS, 2 mmol/L l-glutamine, and 1% penicillin and streptomycin. PBMCs were rested overnight and treated with PBS-vehicle or 100 ng/mL LPS and 10 ng/mL of recombinant IFN-γ (R&D Systems) for 6 hours. After stimulation, PBMCs were lysed in RLT (QIAGEN), snap-frozen, and stored at −80 °C. RNA was isolated and subject to RNA-sequencing (RNA-seq) as described.

### Metabolomics and Metabolite Measurements

Isolated human monocytes were cultured at a density of 3.5×10^5^ cells per sample in complete 5 mmol/L glucose DMEM, media supplemented with glucose to make a final concentration of 20 mmol/L glucose, or media with 15 mmol/L mannitol in 5 mL fluorescence-activated cell sorting tubes for 12 hours. Cell lysates were prepared by extraction with ice-cold methanol for 20 minutes on dry ice and snap-frozen in liquid nitrogen. On the day of analysis, samples were vortexed and then centrifuged at 10 ,000*g* for 10 minutes at 4 °C. A pooled quality control sample was prepared by mixing 60 µL each sample extract and analyzed periodically throughout the overall analytical run to confirm method performance. Samples were filtered with a 0.1-µm Ultrafree-MC centrifugal filter (5000*g*, 3 minutes) and then transferred to liquid chromatography–mass spectrometry vials. Nontargeted metabolomics analyses were performed as previously described.^27^ Briefly, samples were analyzed on a Thermo Ultimate 3000 ultrahigh-performance liquid chromatography coupled to a Q-Exactive Orbitrap using polarity switching (positive-negative) acquisition operating at a mass resolving power of 70 000 full width at half-maximum. Hydrophilic compounds were chromatographically separated on a Merck Sequant ZIC-HILIC column (150×4.6 mm, 5-μm particle size) and hydrophobic compounds using a Thermo Accucore aQ RP C18 column (150×2.1 mm, 2.7-μm particle size). Metabolites were identified using an in-house database.^27^ Metabolite Set Enrichment and Metabolic Pathway Analysis was performed on all metabolite measurements using MetaboAnalyst (version 4.0).

For BMDM and HSC metabolite measurements, cells were cultured as previously described at a density of 5×10^5^ per well, and culture supernatants were run on the ABX Pentra 400 (Horiba) to measure glucose (ABX Pentra Glucose PAP CP, Horiba, UK) and lactate (ABX Pentra Lactic Acid) levels. Measurements were normalized to glucose and lactate concentrations present in control complete cell culture media that was incubated under the same conditions but not in the presence of cells. BMDM cell lysates were used to measure succinate levels (Succinate Assay Kit, Abcam, UK) according to the manufacturer’s instructions.

### ATAC-seq Analysis

Control or diabetic isolated HSCs or BMDMs, unstimulated or stimulated with IL-1β or LPS+IFN-γ for 2 hours, respectively, in 5 mmol/L glucose DMEM were used for ATAC-seq analysis according to a previously described protocol.^28^ Briefly, nuclei from 50 000 HSCs or 75 000 BMDMs per replicate were isolated, lysed on ice, and immediately put through transposition reaction using Tn5 transposase and TD buffer (Illumina, US) for 30 minutes at 37 °C. Library amplification (NEBNext High Fidelity 2× PCR master mix, New England Biolabs, US) was followed by SPRI size selection to exclude fragments >1200 bp. DNA concentrations were measured with a Qubit fluorometer (Life Technologies), and library size distribution was assessed with the Bioanalyser DNA High-Sensitivity Chip (Agilent, UK). Library amplification was performed with custom Nextera primers.^28^ Libraries were sequenced by the Biomedical Sequencing Facility at CeMM using the Illumina HiSeq 3000/4000 platform and the 50-bp single-read configuration.

Reads were controlled for quality and trimmed of any remaining adapters. Reads were then aligned to the mouse genome (Mm10) with Bowtie (version 2.2.6) in “sensitive-local” mode. Aligned reads were filtered to remove low-quality matches (mapping quality ≤10) or reads mapping to mitochondrial sequences. Alignment start positions were shifted in a strand-aware manner (4 bp, −5bp,^28^) before peak calling with MACS2 (version 2.1.1^29^). Differentially bound regions were identified with DiffBind (version 2.4.8^30^) with peak centering (summits=100) and edgeR.^31^

Pathway analysis was then performed on peaks enriched in diabetic samples (false discovery rate [FDR] ≤0.1) by inputting the peak chromosome location into Genomic Regions Enrichment of Annotations Tool (version 3.0).^32^ Differential peak regions were then analyzed with MEME–chromatin immunoprecipitation (ChIP; version 4.12.0),^33^ which performs comprehensive motif analysis to identify any known or unknown binding motifs that are enriched (enriched motifs E<0.05; minimum width, 6; minimum number of sites, 3).

### Runt-Related Transcription Factor 1 Target Identification

MEME-ChIP of ATAC-seq regions identified Runt-related transcription factor 1 (RUNX1) as a potential regulator in both IL-1β and unstimulated diabetic HSCs. MEME GOMo (version 4.12.0)^33^ was used to scan mouse and human promoters (−1000, +200) for the consensus RUNX1 motif (MA002.2). In addition to Gene Ontology enrichment analysis, GOMo returns a list of genes identified as targets. These hits were restricted to conserved promoters bound in both mouse and human with empirical *P* values <0.05 or 0.01. Mouse Genome Informatics identifiers were converted to Mouse Ensembl identifications on the Mouse Genome Informatics website and then to Human Ensembl identification and gene symbol using the Ensembl BioMart interface. For values of *P*<0.05, 1300 target genes were converted, of which 1251 were represented on the arrays; for values of *P*<0.01, there were 355 converted and 347 on the arrays. Overrepresentation of RUNX1 target genes among the DE genes was assessed by the Fisher exact test as implemented in R. The pheatmap package (version 1.0.8) was used to generate the heat maps, with row-normalized log2 values shown for DE genes. Both genes and samples were clustered by Pearson correlation.

### RNA-seq Analysis

Control or diabetic BMDMs (n=6), cultured in 5 mmol/L and stimulated with LPS+IFN-γ for 6 hours or unstimulated control cells, were used for RNA-seq analysis. Briefly, RNA was isolated with the mirVana mRNA/miRNA Isolation Kit (ThermoFisher Scientific, UK), according to the manufacturer’s instructions. The amount of total RNA was quantified with the Qubit Fluorometric Quantitation system (Life Technologies), and the RNA integrity number was determined with the Experion Automated Electrophoresis System (Bio-Rad). RNA-seq libraries were prepared with the TruSeq Stranded mRNA LT sample preparation kit (Illumina, UK) using both Sciclone and Zephyr liquid handling robotics (PerkinElmer, UK). Library concentrations were quantified with the Qubit Fluorometric Quantitation system (Life Technologies, UK), and the size distribution was assessed with the Experion Automated Electrophoresis System (Bio-Rad, US). For sequencing, samples were diluted and pooled in equimolar amounts and sequenced on Illumina HiSeq 3000/4000 instruments in 50-bp single-read configuration by the Biomedical Sequencing Facility at CeMM. Base calls provided by the Illumina Real-Time Analysis software were subsequently converted into binary alignment map format (Illumina2bam) before demultiplexing (BamIndexDecoder) into individual, sample-specific binary alignment map files via Illumina2bam tools (1.17.3). Libraries were prepared and sequenced by the Biomedical Sequencing Facility at CeMM.

Reads were aligned to the mouse genome (GRCm38/mm10) using STAR (version 2.5.3a^34^) in gene counting mode (quantMode GeneCounts) with Gencode (version M16) annotations (from which megatranscript Gm20388 was removed to avoid overlapping intervening genes). Counts were loaded into R (version 3.3.3) and analyzed with edgeR (version 3.16.5^35^) after removing transcripts with <0.5 mapped reads per million sequenced in all samples (13 568 transcripts retained). Genes with adjusted values of *P*<0.05 and fold change >1.5 were deemed to be DE. DE tables and gene lists are included as Excel File II in the Data Supplement (unstimulated) and Excel File III in the Data Supplement (LPS+IFN-γ stimulated). The edgeR function cpm was used to log-normalize counts (Excel File IV in the Data Supplement) for heat maps and other plots. Heat maps were generated with the R package pheatmap (version 1.0.8^36^). Overrepresentation of RUNX1 target genes analysis among DE genes in diabetic compared with control LPS+IFN-γ samples was performed as previously described for the human array data.

### ChIP Sequencing

A total of 1×10^6^ cells/mL were fixed with 1% formaldehyde at room temperature for 10 minutes, washed with PBS, and stored at −80 °C. Cells were sheared with the Chromatin EasyShear Kit–Low SDS (Diagenode, Belgium) and sonicated with a Bioruptor Pico sonication device (catalog No. B01080010, Diagenode, Belgium) according to the manufacturer’s recommendations. The MAGnify Chromatin Immunoprecipitation System (Thermo Scientific) was used to select chromatin fragments using anti–histone H3 (trimethyl K4; ab8580) or anti-histone H3 (acetyl K27; ab4729). Sequencing libraries were prepared with the NEBNext Ultra II DNA Library Prep Kit according to manufacturer’s instructions. In brief, purified ChIP or input DNA was end repaired and A-tailed followed by adaptor ligation. The adaptor-ligated DNA was size selected with SPRI Ampure XP beads (Agencourt) and enriched with Illumina-compatible sequencing primers. The final libraries were purified with SPRI Ampure XP beads to remove adaptor dimers and sequenced by the Biomedical Sequencing Facility at CeMM using the Illumina HiSeq 3000/4000 platform and the 50-bp single-read configuration.

### Bioinformatics

To examine the relationship between chromatin openness and gene expression in unstimulated BMDMs, diabetes versus control ATAC-seq peaks (log2 fold changes) and the mRNA level of the proximate genes (log2 fold changes) were compared by Pearson linear correlation.

Reads from ATAC-seq, H3K4me3 ChIP sequencing (ChIP-seq), and H3K27ac ChIP-seq experiments were visualized in representative samples using the Integrative Genome Browser, with track heights scaled per experiment and cell type to account for systematic differences in read depth.

### Statistical Analysis

All nonsequencing data statistical analysis was performed in GraphPad Prism software. Data were analyzed for normal distribution by Shapiro-Wilk normality test. Comparisons between 2 groups were calculated by 2-tailed Student *t* tests or Mann-Whitney *U* tests for normally or nonnormally distributed data, respectively. Comparisons between multiple groups were calculated by 1-way or 2-way ANOVA analysis with Bonferroni post hoc correction. Data are reported as mean±SD for normally distributed data or median±interquartile range for nonnormally distributed data. Number of replicates and other statistical details can be found in the figure legends. For both in vivo and in vitro experiments, n is the number of individual animals or individual healthy volunteers or patients. Fold changes are calculated as average test condition value divided by average control value.

## RESULTS

### Diabetic Hyperglycemia Alters Cellular Metabolism, Driving Proinflammatory Gene Expression and Function

Aerobic glycolysis increases proinflammatory (M1) gene expression,^37^ and hyperglycemia also promotes M1-associated gene expression.^10,11^ Therefore, we hypothesized that high extracellular glucose drives increased glycolysis, leading to enhanced polarization of macrophages toward an M1 phenotype.

**Figure 1.**
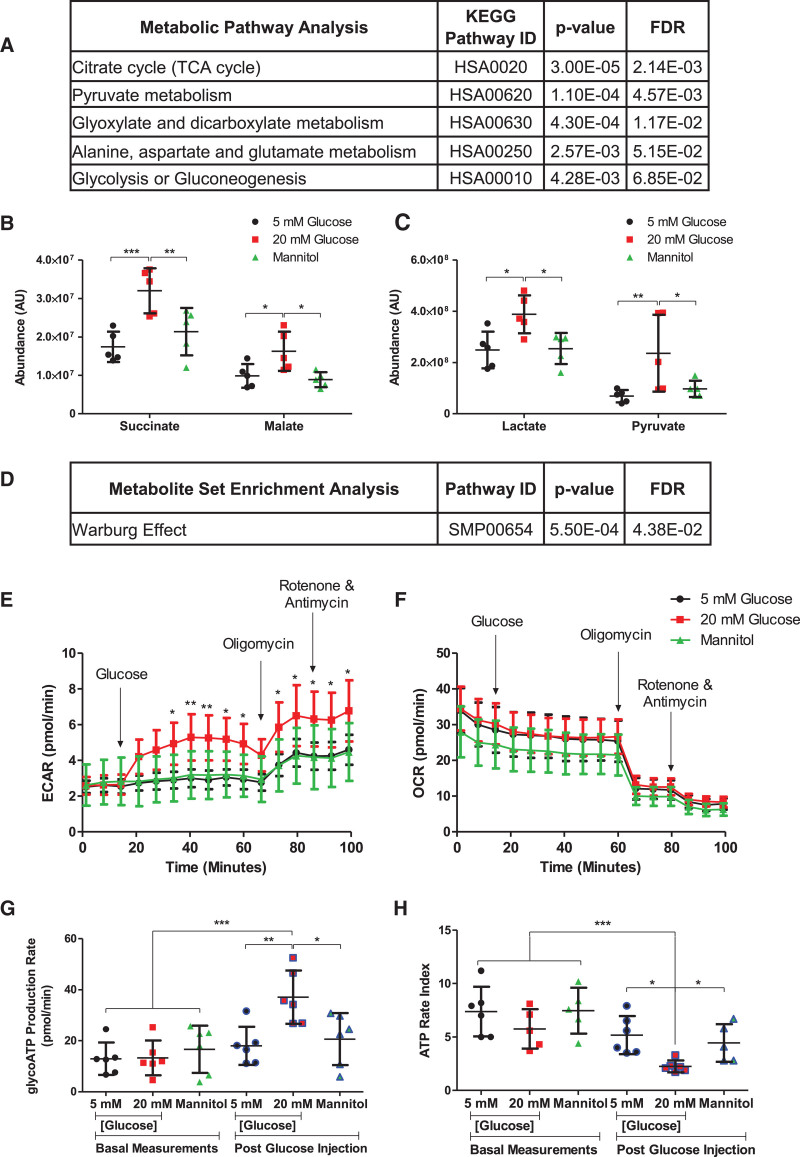
**High glucose alters cellular metabolism**. **A**, Metabolic pathway analysis. Individual measurements for (**B**) succinate, malate, (**C**) lactate and pyruvate and (**D**) metabolite set enrichment analysis (Metaboanalyst) for human monocyte cell lysate metabolomics samples (n=5). Extracellular flux analysis on mouse bone marrow–derived macrophages (Seahorse bioanalyzer; n=6 individual animals) measured (**E**) extracellular acidification rate (ECAR) and (**F**) oxygen consumption rate (OCR) in response to various extracellular glucose, osmotic control (mannitol), and metabolic inhibitor injections. High glucose (**G**) increased glycolytic ATP (glycoATP) production and (**H**) decreased ATP rate index (mitochondrial ATP production rate/glycoATP production rate), indicating a shift to a more glycolytic phenotype. Data analyzed by (**B** and **C**) 2-way ANOVA or (**E–H**) 1-way ANOVA plus Bonferroni post hoc test. All data shown are mean±SD. AU indicates arbitrary units; FDR, false discovery rate; and TCA, tricarboxylic acid. *P<0.05; **P<0.01; ***P<0.001.

Metabolome analysis of primary human monocytes indicated that high extracellular glucose (20 mmol/L) shifted the metabolic profile compared with osmotically matched physiological glucose (5 mmol/L) conditions. Pathway analysis (Figure 1A) revealed that high glucose drove significant changes in the tricarboxylic acid cycle (FDR, 0.002) and pyruvate metabolism (FDR, 0.005), denoted by increased succinate, malate (Figure 1B), lactate, and pyruvate (Figure 1C) levels, respectively, with metabolite set enrichment analysis (Figure 1D) highlighting the Warburg effect (FDR, 0.04).

A more glycolytic (and less oxidative) phenotype in response to high glucose (20 mmol/L) was confirmed in mouse BMDMs, which showed an increase in extracellular acidification rate, increased glycolytic ATP production (2-fold; *P*<0.01), decreased ATP rate index (by 56%; *P*<0.05; Figure 1E–1H), and increased glucose consumption and lactate production (*P*<0.001; Figure Ia and Ib in the Data Supplement). Cultured BMDMs also increased succinate levels in response to high extracellular glucose in both unstimulated (2.5-fold, *P*<0.05) and M1-stimulated states (1.9-fold; *P*<0.001; Figure Ic in the Data Supplement).

This increase in glycolytic rate was prevented by the inhibitor of glycolysis dichloroacetate (Figure Ia and Ib in the Data Supplement).

**Figure 2.**
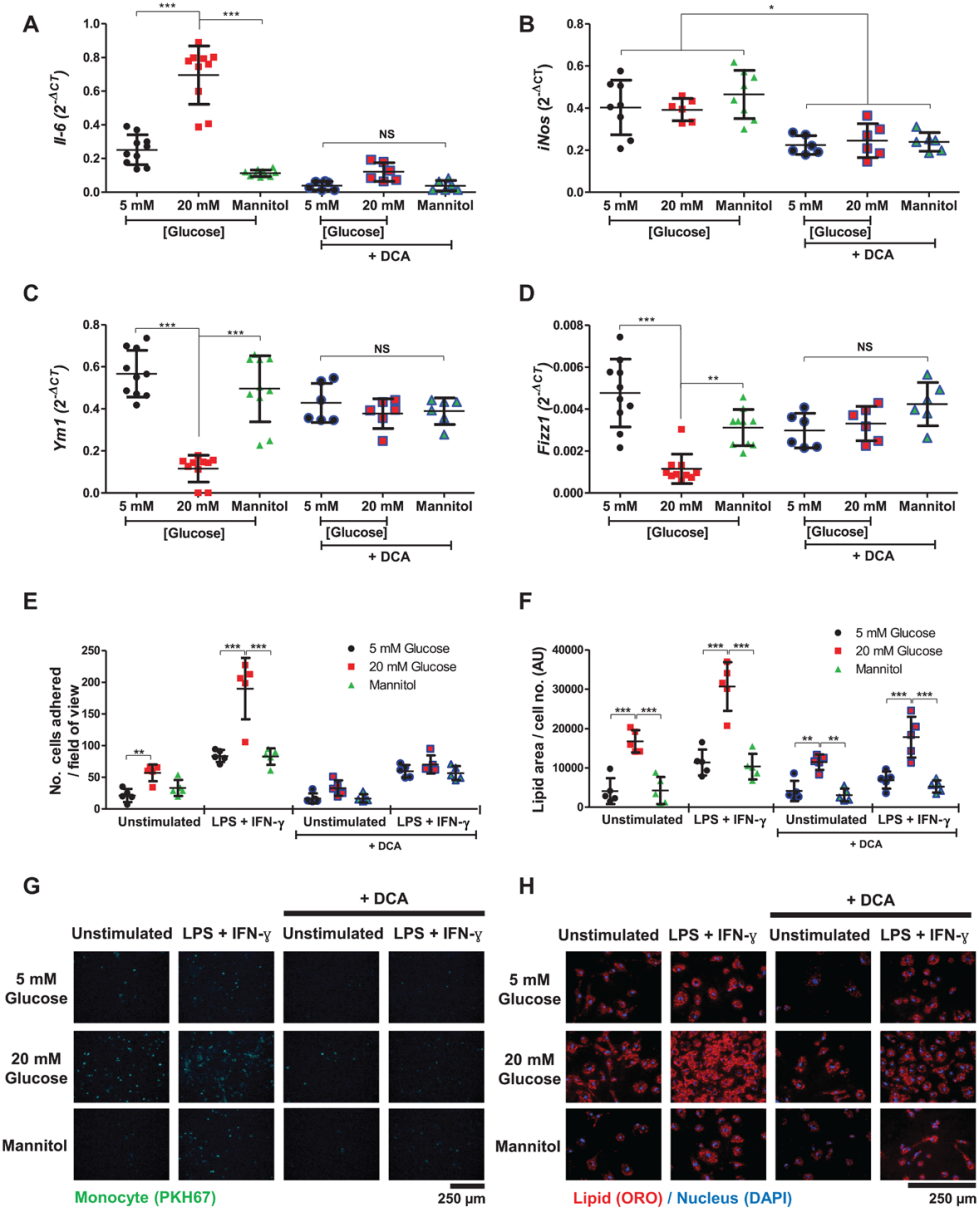
**High glucose alters macrophage gene expression and function.** M1 (lipopolysaccharide [LPS]+interferon-γ [IFN-γ] stimulated) bone marrow–derived macrophage (BMDM) (**A**) *Il-6* and (**B**) *iNos* gene expression in the presence or absence of dichloroacetate (DCA). M2 (interleukin-4 stimulated) BMDM (**C**) *Ym1* and (**D**) *Fizz1* gene expression. **A** through **D**, Quantitative polymerase chain reaction data are normalized to *B2m* expression; n=7 to 10. **E**, Image quantification of mouse monocyte adhesion to endothelial cells (n=5). **F**, Image quantification of acetylated low-density lipoprotein uptake by BMDMs, normalized by cell number per image. Representative (**G**) static adhesion images of fluorescently PKH67-labeled mouse monocytes to endothelial cells and (**H**) Oil Red O (ORO)– and DAPI-stained foam cell images. All data (**A–H**) are shown as mean±SD; (**A–D**) 1-way ANOVA or (**E** and **F**) 2-way ANOVA with Bonferroni post hoc analysis. Each point represents an individual animal (average of 4 images for static adhesion and foam cell formation). AU indicates arbitrary units; NS, nonsignificant. *P<0.05; **P<0.01; ***P<0.001.

In BMDMs, high glucose both promoted proinflammatory M1-associated *Il-6* expression (2.7-fold; *P*<0.001; Figure 2A) and suppressed M2-associated *Ym1* (70%; *P*<0.001) and *Fizz1* (42%; *P*<0.001) expression (Figures 2C and 2D). These bidirectional changes in gene expression were restored in the presence of dichloroacetate (Figure 2A–2D) and 2-deoxy-d-glucose (Figure IIa–IId in the Data Supplement), together indicating that the proinflammatory effects of high extracellular glucose were mediated through changes in glycolysis and were not merely the result of nonspecific changes to overall metabolic activity.

Given its effects on promoting inflammation, we then tested whether high extracellular glucose altered monocyte and macrophage functions relevant to atherogenesis.^38,39^ High glucose increased mouse monocyte adhesion to activated endothelial cells (2.6-fold; *P*<0.001; Figure 2E and 2G) and promoted BMDM uptake of modified LDL to form foam cells (4.1-fold [and 2.7-fold when M1 stimulated]; *P*<0.001 for both; Figure 2F and 2H). The effects on monocyte adhesion were negated entirely by inhibition of glycolysis with dichloroacetate (*P*<0.001; Figure 2E and 2G) and 2-deoxy-d-glucose (*P*<0.001; Figure IIe in the Data Supplement), whereas foam cell formation was decreased by 30% (and by 42% when M1-stimulated; *P*<0.001 for both; Figure 2F and 2H).

### BM-Derived Cells From Diabetic Mice Exhibit Hyperglycemia-Induced Trained Immunity

**Figure 3.**
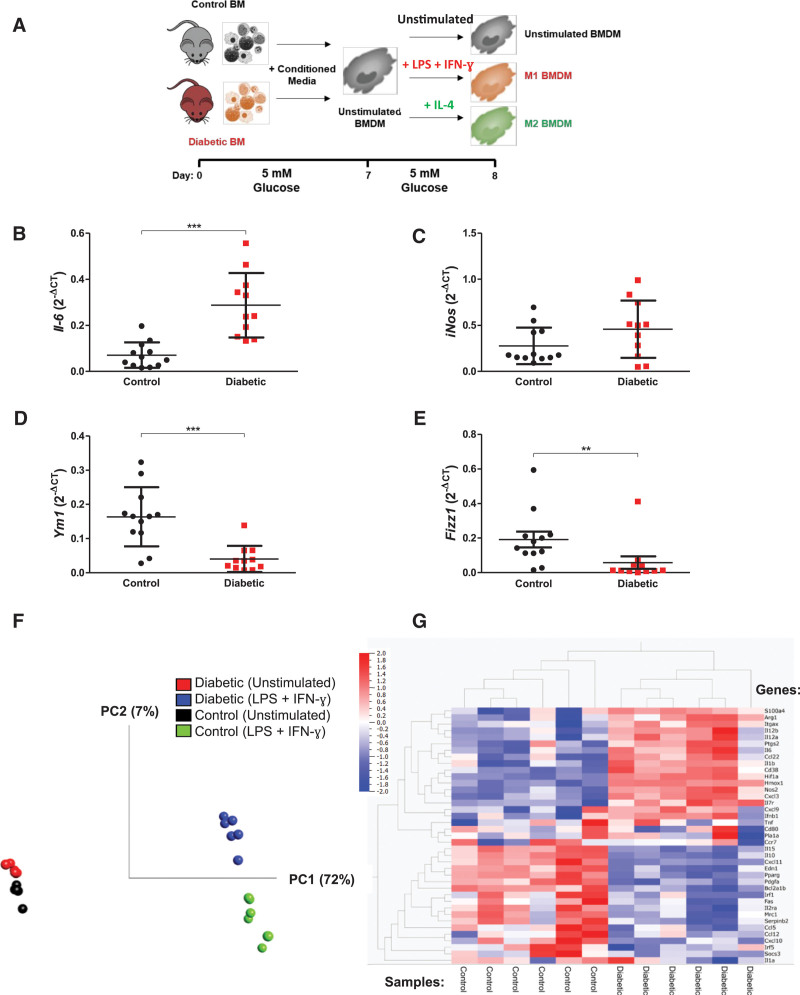
**Bone marrow (BM)–derived macrophages (BMDMs) from diabetic mice manifest hyperglycemic memory in gene expression**. **A**, Schematic of diabetic and control BMDM protocol (n=10–12). M1 gene expression analysis of (**B**) *Il-6* (*P*<0.0001) and (**C**) *iNos*. M2 (**D**) *Ym1* (*P*=0.0003) and (**E**) *Fizz1* (*P*=0.0326) gene expression. **B** through **E**, All quantitative polymerase chain reaction data are normalized to B2m expression, analyzed by the unpaired *t* test. **F**, Principal component (PC) analysis of all control and diabetic BMDM RNA sequencing (RNA-seq) transcript reads (n=6); percent indicates sample variability. **G**, Unsupervised hierarchical clustering shows marked segregation of 39 previously identified M1 and M2 macrophage marker genes in lipopolysaccharide (LPS)+interferon-γ (IFN-γ)–stimulated BMDMs from diabetic or control origin (RNA-seq analysis). IL indicates interleukin.

We next used a mouse model of diabetes to test whether hyperglycemia induced persistent proinflammatory changes in macrophages, even after glucose normalization. We obtained BM from nondiabetic, age-matched control mice and from syngeneic diabetic mice in which hyperglycemia had been induced by streptozotocin and maintained for 6 weeks in vivo (Figure IIIa–IIId in the Data Supplement). Cells were differentiated into BMDMs under physiological glucose (5 mmol/L; Figure 3A). After stimulation, BMDMs of diabetic mouse origin displayed enhanced M1-associated *Il-6* gene expression (4.1-fold; *P*<0.0001; Figure 3B) and, with IL-4 treatment, decreased M2-associated *Ym1* expression (by 75%; *P*=0.0003) and *Fizz1* (by 70%; *P*=0.0326; Figure 3D and 3E). RNA-seq analysis of control and diabetic BMDMs revealed clear delineation based on gene expression profiles, which was further accentuated by LPS and IFN-γ stimulation (Figure 3F). Indeed, when expression levels of a panel of 39 previously identified M1- and M2-associated genes were examined,^9^ BMDMs from control and diabetic origin showed clear segregation after LPS+IFN-γ stimulation (Figure 3G).

**Figure 4.**
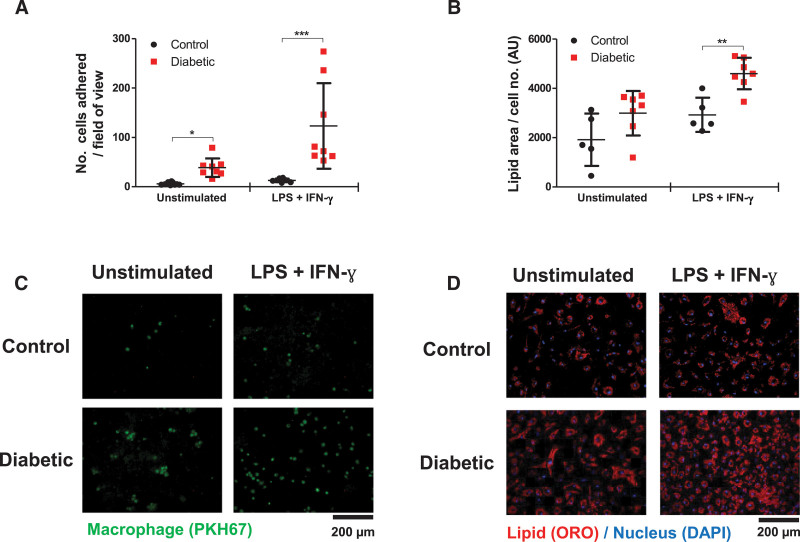
**Bone marrow–derived macrophages (BMDMs) from diabetic mice display altered functions despite glucose normalization.** BMDMs from diabetic mice were differentiated in 5 mmol/L (n=5–8). **A**, Image quantification of BMDM adhesion to endothelial cells (n=8). **B**, Image quantification of acetylated low-density lipoprotein uptake by BMDMs, normalized by cell number per image. Representative (**C**) static adhesion images of fluorescently (PKH67) labeled BMDMs to endothelial cells and (**D**) Oil Red O (ORO)– and DAPI-stained foam cell images. All data are shown as mean±SD, and each point represents an individual animal (average of 4 images for static adhesion and foam cell formation). Data analyzed by 2-way ANOVA with Bonferroni post hoc test. IFN-γ indicates interferon-γ; and LPS, lipopolysaccharide. *P<0.05; **P<0.01; ***P<0.001.

Furthermore, there was increased adhesion to activated endothelium (by 6.3-fold; *P*<0.05; and by 9.4-fold when LPS-stimulated; *P*<0.001; Figure 4A and 4C) and enhanced modified LDL uptake and foam cell formation (LPS stimulated, 1.6-fold; *P*<0.01; Figure 4B and 4D). This array of persistently heightened responses was indicative of hyperglycemia-induced trained immunity.

### Hyperglycemia-Induced Trained Immunity Drives Atherosclerosis

**Figure 5.**
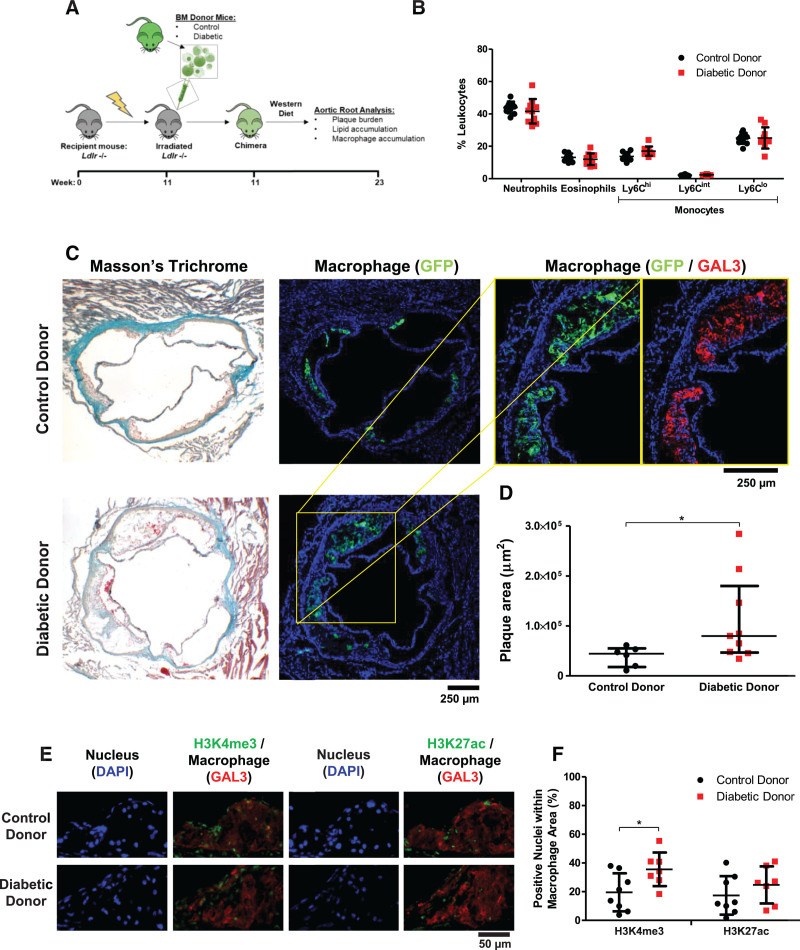
**Diabetic bone marrow (BM) drives atherosclerosis despite prolonged glucose normalization**. **A**, Schematic of BM transplantation experiment. **B**, Flow cytometric analysis of peripheral leukocyte populations in mice after 12 weeks of bone marrow transplantation. **C**, Representative aortic root images from *Ldlr*^−/−^ mice that had received either diabetic donor or control donor CD68-GFP BM. Masson Trichrome stained (left) and immunofluorescence stained for BM transplant–derived macrophages (green indicates green fluorescent protein [GFP]) or all macrophages (red indicates galectin-3 [GAL3]). Image quantification of (**D**) plaque volume (*P*=0.036). Data shown as mean±SD, analyzed by 1-way ANOVA with Bonferroni post hoc test (**B**), or median±interquartile range, analyzed by Mann-Whitney *U* test (**D**); control n=6, diabetic n=9. **E**, Representative aortic root images from *Ldlr*^−/−^ mice that had received either diabetic or control BM transplantation. **F**, Quantification of the percentage of nuclei stained positive for H3K4me3 or H3K27ac within the GAL3 macrophage positive region. Each data point represents an individual animal (average, 6 sections; n= 7 to 10). Shown as mean±SD. One-way ANOVA analysis. BMDM indicates BM-derived macrophages; HSC, hematopoietic stem cell; IFN-γ, interferon-γ; and LPS, lipopolysaccharide. *P<0.05.

Therefore, to test the functional significance of hyperglycemia-induced trained immunity in atherosclerosis, we transplanted BM from normoglycemic control or streptozotocin-induced diabetic donor mice (blood glucose, 33.9±6.9 mmol/L) into atherosclerosis-prone (normoglycemic) *Ldlr*^−/^^−^ recipient mice (Figure 5A). In each case, the donor mice were transgenic for GFP under the control of the human CD68 promoter.^40^ Effective and equivalent engraftment was shown by flow cytometric analysis of PBMCs, confirming the presence of CD45+ GFP+ cells positive for either CD11b or Ly6C (Figure IVa and IVb in the Data Supplement). After 12 weeks on a Western-type diet, there were no differences in blood lipid measurements (Figure IVc and IVd in the Data Supplement) or leukocyte subsets as assessed by flow cytometry (Figure IVe in the Data Supplement).

Mice that had received diabetic BM showed markedly increased atherosclerosis, with a majority having more than the greatest atherosclerosis burden in the nondiabetic recipients (median plaque areas, 44 461 µm^2^ [interquartile range, 18 368–59 597 µm^2^] versus 79 809 µm^2^ [44 745–177 740 µm^2^]; Mann-Whitney, *P*=0.036). These mice also showed an increase in macrophage content of the plaques (*P*=0.052; Figure V in the Data Supplement), lipid-rich necrotic core area (2.8-fold, *P*=0.0076; Figure VIa in the Data Supplement), and proportion lipid-rich necrotic core (2.4-fold, *P*=0.046; Figure VIb in the Data Supplement), but with no differences in collagen content (plaque percent; Figure VIc and VId in the Data Supplement) or smooth muscle cells (plaque percent; Figure VIe and VIf in the Data Supplement).

### Hyperglycemia-Induced Trained Immunity in HSCs Is Driven by Changes in Metabolism Leading to Epigenetic Reprogramming

Given the demonstration that BM from hyperglycemic mice promoted atherosclerosis, along with the persistent proinflammatory gene expression and functional responses found in BMDM in vitro, we reasoned that this memory was a manifestation of trained immunity in BM-derived cells. Trained immunity has been associated with alterations in cellular metabolism that induce long-lasting, chromatin-altering epigenetic marks such as H3K4me3 and H3K27ac.^17,18,41^ In line with this, we found that HSC (lineage negative: removal of mature T cells, B cells, natural killer cells, dendritic cells, monocytes, granulocytes, erythroid cells: CD3−, CD4−, B220−, Ter119, Gr1− cultured in conditions of high extracellular glucose also displayed increased glucose uptake and lactate production, indicating dysregulated glycolysis (*P*<0.001; Figure VIIa–VIId in the Data Supplement), and both H3K4me3 and H3K27ac modifications were increased (Figure VIIIa–VIIIf in the Data Supplement). In the case of H3K4me3 (but not H3K27Ac), this effect was negated with dichloroacetate inhibition (Figure VIIIa, VIIIc, and VIIId in the Data Supplement). These chromatin marks were also consistently increased in HSCs from diabetic mice compared with control HSCs and remained heightened after differentiation into BMDMs using physiological glucose levels (5 mmol/L; Figure VIIIb, VIIIe, and VIIIf in the Data Supplement).

In aortic root plaques, the proportion of macrophage-associated nuclei stained positive for H3K4me3 was higher in mice that had received BM from diabetic donors (1.8-fold, *P*<0.05) compared with control donors (Figure 5E and 5F), which is in line with the corresponding analysis of BMDM in vitro experiments (Figure VIIIb and VIIIe in the Data Supplement). These data point to a direct causal link between increased glycolytic rate and the development of hyperglycemia-induced trained immunity. In addition to metabolic mediators,^17,18^ recent work has implicated the inflammatory cytokine IL-1β in the development of trained immunity.^42^ This is particularly relevant because IL-1β has been identified in diabetes^43^ as an important driver of atherosclerosis and in obesity in which adipose tissue macrophage-produced IL-1β induces proliferation of BM progenitors.^44,45^ Therefore, we hypothesized that IL-1β and hyperglycemia may work synergistically to induce atherosclerosis-relevant epigenetic changes that alter chromatin structure. To interrogate chromatin accessibility, we performed an assay for transposase-accessible chromatin with high-throughput sequencing (ATAC-seq) on HSCs from diabetic or control mice, both with and without IL-1β stimulation^28^ (sample processing summarized in Excel File V in the Data Supplement). HSCs from a diabetic origin showed differential chromatin profiles (summarized in Excel File VI in the Data Supplement) in response to IL-1β exposure, with 530 differential peaks (FDR ≤0.1) of which 50% exhibited increased accessibility in diabetic cells. To explore the biological relevance of this set of noncoding genomic regions (with emphasis on putative cis-regulatory function), the regions of chromatin with increased accessibility in diabetic cells were assigned to the 2 nearest genes (within 1000 kb) and analyzed with the Genomic Regions Enrichment of Annotations Tool.^33^ This pathway analysis revealed augmentation of several inflammation-related pathways, including leukocyte activation (3.3-fold; FDR, 0.0005), and genes associated with LPS tolerization in macrophages (2.7-fold; FDR, 0.0009).

To understand whether high glucose in isolation could also induce chromatin modification, we used ATAC-seq to look at HSCs from control and diabetic mice in the absence of IL-1β stimulation. Unstimulated HSCs displayed 357 differential peaks (FDR ≤0.2), of which 51% had increased accessibility in cells from a diabetic origin compared with control, with the majority in intronic (48%) or intergenic (21%) regions.

**Figure 6.**
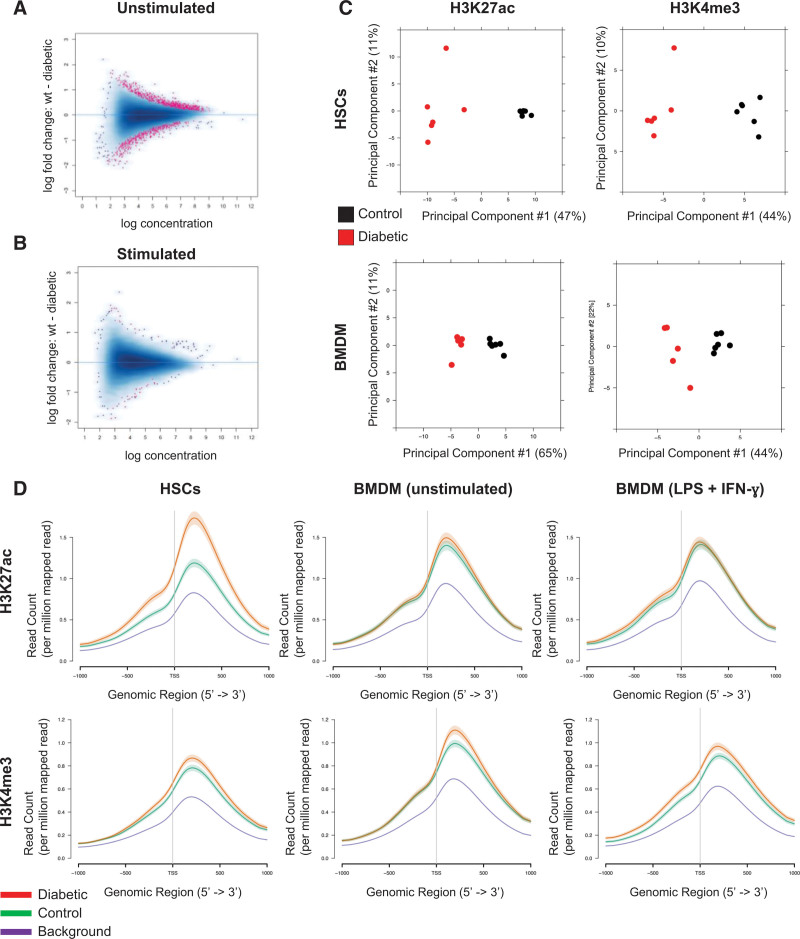
**Diabetes alters levels of H3K4me3 and H3K27ac and chromatin accessibility.** MA plot of all assay for transposase accessible chromatin sequencing chromatin reads in (**A**) unstimulated or (**B**) stimulated bone marrow–derived macrophages (BMDMs), where peaks with differential accessibility (false discovery rate <0.05, fold change >1.5) are highlighted in pink (1047 unstimulated, 40 stimulated, n=6). **C**, Variable regions of H3K4me3 and H3K27ac quantified by chromatin immunoprecipitation sequencing in hematopoietic stem cells (HSCs) and BMDMs from control versus diabetic mice (n=6 per group). **D**, Histogram of the average coverage of H3K27ac and K3K4me3 in HSCs, unstimulated BMDMs, and proinflammatory stimulated BMDMs (lipopolysaccharide [LPS]+interferon-γ [IFN-γ]) at transcription start sites±1 kb of protein-coding genes proximate to regions of increased accessibility in unstimulated BMDMs (vs controls; n = 405) and compared with background levels in nonselected regions.

Given these findings in HSCs, we next examined chromatin accessibility and gene expression in their derivative BMDMs. We performed ATAC-seq and RNA-seq on BMDMs from control and diabetic mice under unstimulated or proinflammatory stimulated (LPS+IFN-γ) conditions. BMDMs from diabetic mice showed differential chromatin accessibility under unstimulated conditions, with 1047 differential regions (FDR <0.05; Figure 6A) but only 40 differentially open regions (FDR <0.05) after stimulation (Figure 6B). In contrast, unstimulated BMDMs from diabetic mice differentially express 632 genes (324 upregulated expression and 308 downregulated expression; Excel File II in the Data Supplement) compared with unstimulated control BMDMs, but this increases to 1348 genes (802 increased and 546 decreased; Excel File III in the Data Supplement) on stimulation (FDR <0.05; fold change >1.5). Taken together, the ATAC-seq and RNA-seq data suggest that diabetes primed macrophages for an exaggerated response to the proinflammatory stimulus.

We further undertook an unbiased examination of the relationship between differential gene expression and altered chromatin accessibility in the diabetic BMDMs. We annotated the nearest or overlapping gene for each region of differentially accessible chromatin in BMDMs (M0 state) from diabetic mice versus controls. For genes within this set that were also DE (cutoff FDR, 0.1; log fold change >0.5 for each), we compared the fold change for differential ATAC-seq peak versus fold change for DE. Examined as continuous variables, there was a strong correlation between alterations in chromatin and expression of proximate genes (*R*=0.65; *P*<1×10^−6^).

Furthermore, to ascertain the nature of the diabetes-induced chromatin modifications, we undertook ChIP-seq in HSCs and BMDMs from control versus diabetic mice (n=6 per group). Diabetic HSCs and BMDMs were clearly distinguishable from control samples on the basis of the quantification of both H3K27ac and H3K4me3 histone modifications (Figure 6C), and we confirmed these results by Western blot analysis (Figure VIII in the Data Supplement). To integrate the ChIP-seq and ATAC-seq data, we examined the distribution of H3K4me3 and H3K27ac histone modifications in relation to the regions of open chromatin that were identified by ATAC-seq in unstimulated diabetic BMDMs. A majority of these regions were identified as enhancers, located >1 kb from annotated transcription start sites. We focused on genes in proximity to these enhancers because their gene promoter regions (transcription start sites±1 kb) represent the most likely regulatory targets. ATAC-seq had identified 405 regions of differentially open chromatin (*P*<0.05) in BMDMs derived from diabetic mice versus control mice under basal conditions. In the HSCs from diabetic mice, there was a marked increase in promoter-associated H3K27ac compared with control HSCs (Figure 6D). These differences diminished in BMDMs (Figure 6D), in part because of increased H3K27ac in the nondiabetic origin cells after differentiation. However, the H3K4me3 mark was increased at these promoters in HSC, with identifiable differences persisting into differentiated macrophages, including after cytokine stimulation (Figure 6D). This pattern of transient H3K27ac and persistent H3K4me3 marks is consistent with our findings in both tissue culture (Figure VIII in the Data Supplement) and immunohistochemistry in mouse plaque macrophages (Figure 5E and 5F).

**Figure 7.**
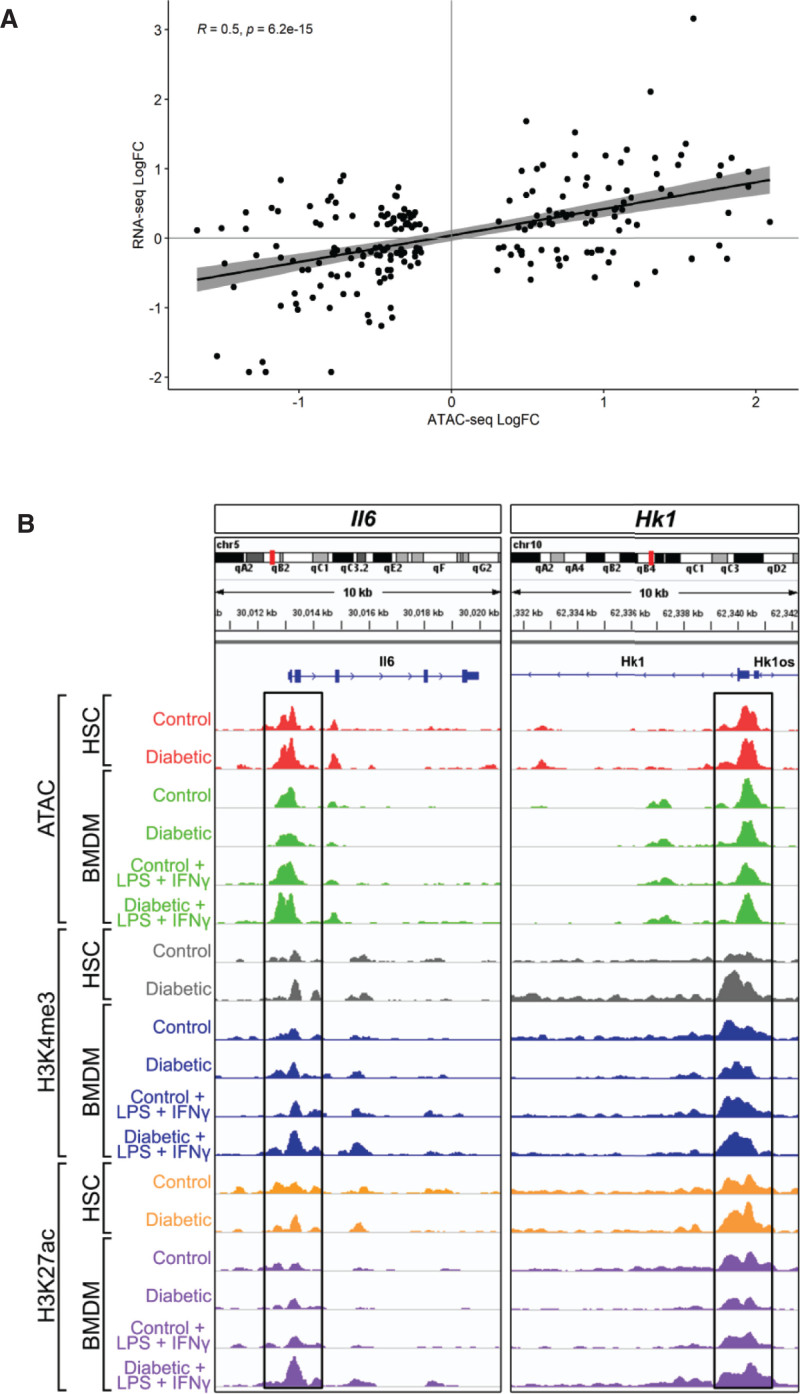
**Chromatin modifications in diabetic mice**. **A**, Comparison (log2 fold change [FC], diabetes vs control) of assay for transposase accessible chromatin sequencing (ATAC-seq) peaks and the mRNA level of the proximate genes from unstimulated bone marrow–derived macrophages (BMDMs). An adjusted false discovery rate cutoff of *P*<0.05 was applied to ATAC-seq regions and RNA sequencing (RNA-seq) genes. **B**, Integrative Genome Browser track plots for *Il-6*) and *Hk1* showing ATAC-seq, H3K4me3 chromatin immunoprecipitation sequencing (ChIP-seq), and H3K27ac ChIP-seq reads from representative samples of hematopoietic stem cells (HSCs), unstimulated BMDMs and proinflammatory stimulated BMDMs (lipopolysaccharide [LPS]+interferon-γ [IFN-γ]) from control and diabetic mice. Regions at transcription start sites±1 kb containing peaks are highlighted by black boxes.

Areas of differentially open chromatin identified by ATAC-seq were highly significantly associated with changes in the transcription of the proximate genes. Furthermore, the quantification of the difference in chromatin accessibility (diabetes versus control) and direct comparison with the change in mRNA expression levels of the proximate gene were highly correlated (*R*=0.5; *P*=6.2e^−15^; Figure 7A). To examine these effects at relevant genomic loci, we combined the ATAC-seq, H3K4me3 ChIP-seq, and H3K27ac ChIP-seq data at the promoters of 2 genes previously implicated in glycolysis-induced trained immunity and with products involved in driving inflammation (*Il-6*) and glucose metabolism (*Hk1*). These genes exhibited regions of accessible chromatin at the transcription start sites, with associated H3K4me3 and H3K27ac peaks at these genomic loci (Figure 7B).

**Figure 8.**
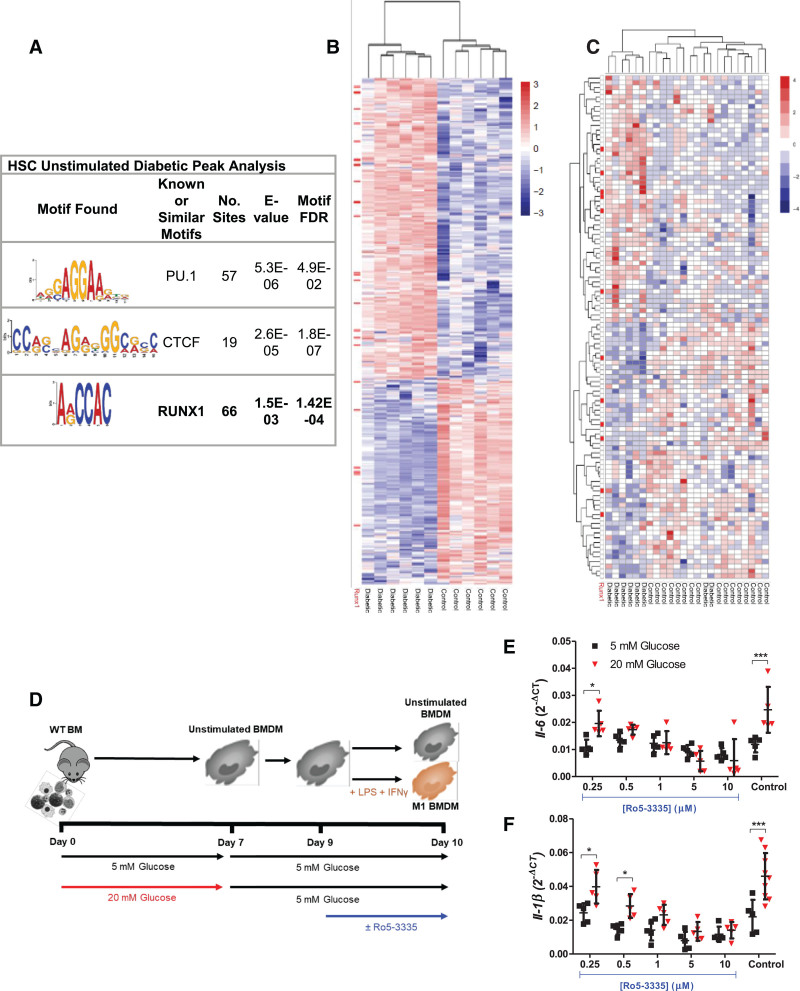
**Role for RUNX1 hyperglycemia-induced trained immunity.** Top 3 transcription factor motifs identified in assay for transposase accessible chromatin sequencing (ATAC-seq) peaks differentially enriched (**A**) unstimulated diabetic HSC (n=3) compared with unstimulated control hematopoietic stem cells (HSCs; n=4). Unsupervised hierarchical clustering of differentially expressed genes from RNA sequencing (RNA-seq) analysis of (**B**) diabetic (n=6) or control (n=6) bone marrow (BM)–derived macrophages (BMDMs) stimulated with lipopolysaccharide (LPS)+interferon-γ (IFN-γ). Unsupervised hierarchical clustering of differentially expressed genes from (**C**) diabetic (n=8) or control (n=16) laser capture microdissected human carotid plaque macrophage samples. **B** and **C**, Runt-related transcription factor 1 (Runx1) target genes (RUNX1) (*P*<0.05 stringency) are highlighted in red to the left of the heat map. Wild-type (WT) BM was differentiated into BMDMs in physiological (5 mmol/L) or high (20 mmol/L) glucose, rested for 2 days in 5 mmol/L glucose, and then stimulated in 5 mmol/L glucose±varying concentrations of Ro5-3335, (**D**) as illustrated. M1 gene expression of (**E**) *Il-6* and (**F**) *Il-1β* assessed by quantitative polymerase chain reaction; data normalized to *B2m* expression. Data displayed as mean±SD, 2-way ANOVA with Bonferroni post hoc. Sample n=5 individual animals. FC indicates fold change. *P<0.05; ***P<0.001.

To gain insight into which transcription factors might be implicated as mediators of this pattern of hyperglycemia-induced trained immunity, Motif Enrichment Analysis^33^ was performed on regions of chromatin with increased accessibility under diabetic conditions. Significantly enriched transcription factor motifs notably included RUNX1 (FDR, 0.0001), which has previously been associated with immunologic memory,^46^ as well as PU.1 (FDR, 0.04) and CCCTC-binding factor (FDR, 1.8×10^−7^; Figure 8A). Having implicated the transcription factor RUNX1^47^ on the basis of the accessibility of binding motifs, we investigated whether RUNX1 target genes were significantly overrepresented among DE genes in LPS+IFN-γ–stimulated BMDMs. This analysis identified 95 of 1348 DE genes as RUNX1 targets (*P*<0.05; odds ratio, 1.3; RUNX1 binding site *P*<0.05; Figure 8B and full differential gene list with RUNX1 target genes marked in Excel File VII in the Data Supplement), confirming its relevance in these disease-modifying cells. To test for RUNX1 involvement in human disease, we interrogated gene expression of macrophages obtained from carotid atherosclerotic plaques of patients with or without diabetes (Excel File I in the Data Supplement). LCM was used to isolate macrophages from human carotid atherosclerosis plaques in patients undergoing clinically driven endarterectomy. Figure 8C shows the DE genes between diabetic and nondiabetic plaque macrophages (FDR <0.2) and highlights in red the RUNX1-associated genes (further detailed in Figure IX in the Data Supplement). RUNX1 target genes were significantly overrepresented among the DE genes, even more so when target binding sites were more stringently restricted: At a binding site *P*<0.05, 12 of 106 DE genes were among the 1251 RUNX1 targets (*P*=0.03; odds ratio, 2.0). These target genes include diabetes-induced DE of atherosclerosis relevant genes such as *Traf3ip3* (log fold change, 0.56; FDR, 0.02),^48^ *Crnkl1* (log fold change, 0.63; FDR, 0.04),^49^ and *Pla2g5* (log fold change, 0.16; FDR, 0.1).^50^

To establish whether the human plaque macrophage functional profile matched that of the mouse macrophages, we compared it with the set of 39 M1 and M2 genes with an expression that had been measured in stimulated BMDMs from diabetic versus control mice (Figure 3G). These were summarized by positive or negative log fold change (diabetic versus control) and compared with the equivalent changes in the human plaque macrophages of patients with type 2 diabetes (versus control). A Fisher exact test showed a significant relationship between the mouse BMDM and human plaque macrophage data sets for genes upregulated or downregulated in diabetic versus control subjects (*P*=0.0155; odds ratio, 6.83).

Having shown a hyperglycemia-induced trained immunity phenotype in mouse HSCs and BMDMs and confirmed shared transcriptional features with macrophages from human plaques, we hypothesized that circulating human blood cells from patients with diabetes might also show evidence of priming. We obtained PBMCs from patients with type 2 diabetes and control subjects without diabetes matched for age and body mass index (Figure X in the Data Supplement). After isolation, PBMCs were restored to normal glucose conditions (5 mmol/L) in tissue culture for 24 hours. Comparing transcriptomes in basal conditions and after stimulation with LPS+IFN-γ, we found that the cells derived from patients with diabetes showed DE of >3000 genes compared with the matched nondiabetic controls. Gene set enrichment analysis of altered genes showed positive enrichment in stimulated diabetic PBMCs (versus controls) for Hallmark pathways, including inflammatory response (*P*=1.70×10^−5^) and IL-6 JAK STAT3 signaling (*P*=0.0221), and reactome pathways, including reactive oxygen species and reactive nitrogen species production (*P*=0.0049), IL-α/IL-β signaling (*P*=0.0110), and Toll-like receptor 1/2 cascade (*P*=0.0033). These pathways demonstrate that these peripheral cells also showed priming characteristics similar to those that we report in BMDMs from diabetic mice (Figure 3G).

Last, to further explore a possible causal role for RUNX1 in hyperglycemia-induced trained immunity responses, BMDMs that had previously been differentiated in physiological or high glucose were restored to 5 mmol/L glucose and then stimulated in the absence or presence of the pharmacological RUNX1-specific inhibitor Ro5-3335^51^ (Figure 8D). Cells differentiated in high glucose displayed increased expression of *Il-6* (2-fold, *P*<0.001) and *Il-1β* (2.1-fold, *P*<0.001), despite normalization of glucose levels (Figure 8E and 8F and Figure XIb and XIc n the Data Supplement). Ro5-3335 normalized heightened *Il-6* and *Il-1β* expression in a dose-dependent manner (Figure 8E and 8F), with the starting effective dose (0.5 µmol/L) being equal to the Ro5-3335 IC_50_ value.^52^

## Discussion

High rates of cardiovascular complications persist in patients with diabetes despite intensive glucose control.^52^ Here, we provide evidence that hyperglycemia induces trained immunity in HSCs and macrophages and that this markedly exacerbates atherosclerosis. We show that hyperglycemia induces persistent modifications in BM HSCs by driving aerobic glycolysis, leading to particular patterns of chromatin accessibility, with evidence for a proinflammatory priming state that persists into differentiated macrophages. Our findings implicate transcription factors, notably RUNX1, as mediators of trained immunity. Pharmacological inhibition of RUNX1 removed these manifestations of hyperglycemia-induced trained immunity in vitro.

Changes in cellular metabolism can induce trained immunity, a term that denotes the innate immune memory that can develop after brief stimulation with microbial products or endogenous atherogenic substances, including oxidized LDL and lipoprotein(a).^53^ For instance, trained immunity induced by β-glucan, a cell-wall component of *Candida* *albicans*, is mediated through activation of the dectin-1–Akt–mTOR–HIF-1α pathway, which causes a switch from oxidative phosphorylation to aerobic glycolysis (Warburg effect).^18^ Indeed, several metabolites from glycolysis and the tricarboxylic acid cycle act as cofactors for histone-modifying enzymes, which in turn determine chromatin accessibility; for example, histone acetyltransferase and deacetylases require acetyl-CoA and NAD+, whereas demethylases such as Tet and JmjC use α-ketoglutarate as a cofactor.^19,54^ Recently, the metabolic regulation of macrophage inflammatory gene expression has been directly linked to the histone modification lactylation derived from increased lactate levels.^55^

The direct links between glucose metabolism and epigenetic modifications, coupled with observations suggesting hyperglycemic memory in patients with diabetes, led us to the hypothesis that hyperglycemia itself may drive changes in cellular metabolism, leading to innate immune memory in diabetes. In the case of β-glucan, increased aerobic glycolysis in macrophages resulted in increased histone 3 Lys4 trimethylation (H3K4me3) and histone 3 Lys27 acetylation (H3K27ac). Both of these chromatin modification markers were increased in the context of high glucose-driven glycolysis. As demonstrated here and in previous studies, there was enrichment of H3K4me3 on the promoters of genes related to inflammation (eg, *Il-6*) and glucose metabolism (eg, *Hk1*).^16,17^ The tricarboxylic acid cycle intermediates succinate and fumarate are reported to act as antagonists of histone and DNA demethylases,^54^ and consistent with this, we also found that the tricarboxylic acid cycle metabolites succinate and malate are increased in hyperglycemic cells.

Mitroulis et al^20^ showed that, in the context of β-glucan–induced innate immunity in myeloid cell progenitors, cytokines can mediate trained immunity. Specifically, inhibition of IL-1β with the receptor antagonist anakinra inhibited β-glucan–induced trained immunity. Western diet has also been shown to induce epigenetic reprogramming in BM progenitor cells, leading to an inflammasome-mediated trained immunity, implicating IL-1β signaling pathways in trained immunity.^56^ Given that IL-1β mediates multiple pathological processes in diabetes,^44^ we examined whether IL-1β would augment the effects of high glucose in the development of trained immunity. Although diabetes alone showed effects on chromatin accessibility (with motif analysis implicating PU.1, RUNX1, and CCCTC-binding factor), the effects of IL-1β, in the context of cells that were primed by diabetes, was even more pronounced (and implicated ERG, ZNF263 [zinc finger protein 263], and RUNX1). This suggests a hierarchical relationship in which macrophages were primed by hyperglycemia but manifest more florid changes in response to a second stimulus.^43^ Indeed, it is well recognized that changes in chromatin states at promoters can underlie complex functional interactions between different macrophage-activating stimuli. For instance, although IFN-γ is unable to activate many LPS-inducible genes, it can induce histone acetylation and chromatin remodeling at their promoters, thereby priming the cell for future activation in response to LPS.^57^

Motif analysis at sites of open chromatin detected an enrichment in RUNX1 binding sites. From a functional perspective, RUNX1 is required for the generation and maintenance of HSCs and the differentiation of diverse lineages.^58^ In monocytes and macrophages, RUNX1 cooperates with PU.1 (also implicated here) to regulate macrophage colony-stimulating factor (colony stimulating factor-1) receptor, which is essential for the survival, differentiation, and expansion of macrophages.^59^ Of relevance to the functional memory effects that we observed in monocyte adhesion, RUNX1 is also an important controller of monocyte-endothelium interactions through regulation of lymphocyte function-associated antigen 1/CD11a.^31^ Finally, Toll-like receptor 4–mediated inflammation and RUNX1 overexpression increase the production of Toll-like receptor 4–induced IL-6 and IL-1β through RUNX1 binding to the nuclear factor-κB subunit p50, synergizing as a transcriptional coactivator.^60^ Taken together, there is evidence for strong convergence of RUNX1 function in macrophages toward regulating processes that are important in atherogenesis. We therefore cross-referenced genes under putative control of RUNX1 with the transcriptional profiles of cells derived from mouse BMDMs and in macrophages from a relevant human in vivo setting. Human atherosclerotic plaque macrophages and BMDMs, in the presence of diabetes, showed DE of RUNX1-regulated genes, including several previously linked to inflammation and atherosclerosis.^49,50^ Our work extends that of Alrdahe and colleagues,^61^ who recently showed that diabetes-derived human macrophages cultured for 6 days in vitro maintain a proinflammatory priming and hyperpolarize to a proinflammatory phenotype when stimulated with LPS and IFN-γ or tumor necrosis factor-α.

It is interesting that some of these RUNX1 target genes display increased expression in plaque macrophages from patients with diabetes, namely CRNKL1 and PLA2G5, whereas others, like ZNF32 (zinc finger protein 32) and NUP214 (nucleoporin 214), displayed lower expression in diabetic versus nondiabetic plaque macrophages. Although this may superficially seem inconsistent, RUNX1 has been demonstrated to both promote gene expression and act as a transcriptional repressor.^62^ This occurs primarily through the recruitment of histone deacetylases such as HDAC1 and HDAC6^63^ and histone methyltransferases.^62^ Moreover, the physical interactions of RUNX1 with chromatin can contribute to the identity of cell progeny. For instance, disruption of the RUNX1 interaction during mitosis leads to epithelial-to-mesenchymal transition.^64^ Pharmacological inhibition of RUNX1 with Ro5-3335 normalized the altered inflammatory gene expression associated with in vitro hyperglycemia-induced trained immunity. The targeted intervention against RUNX1 provides further evidence of the involvement of RUNX1 in the downstream effects of hyperglycemia-induced trained immunity in macrophages.

Induction of hyperglycemia-induced trained immunity in peripheral macrophages alone could have driven disease progression, but given the long-term effects of trained immunity and the relatively short life span of peripheral myeloid cells, we reasoned that trained immunity was also likely to have been induced at the level of HSCs, as has recently been demonstrated for β-glucan and for Western-type diet.^20^ Although previous studies have typically related epigenetic changes in trained immunity to an in vitro phenotype (eg, cytokine production), we show how trained immunity directly drives the progression of atherosclerosis, an important pathology with an integral inflammatory component. Transplanting BM from diabetic mice to normoglycemic atherosclerosis-prone mice had marked effects on lesion progression and was associated with increased lipid-rich necrotic core, consistent with the aggressive form of atherosclerosis seen in diabetes in humans.^65^

This study is built around examination of the effects of high glucose/hyperglycemia. To allow us to characterize the long-term effects of hyperglycemia, the cardinal features of diabetes, we used the streptozotocin mouse model of diabetes rather than a type 2 diabetes mouse model, which might have incorporated more nuanced features of that syndrome but would effectively confound studies on the effects of glucose. There are materially important differences between type 1 and type 2 diabetes. However, hyperglycemia is common to their diagnosis, drives cardiovascular risk in each, and has been the mainstay target of treatments and monitoring for both types of diabetes for many years. We expect that the newly identified hyperglycemia-induced trained immunity reported here will be elaborated in the future to take into account more specific modulation in relation to many other potential influences (eg, dietary sugar,^66^ patterns of glucose elevation,^67^ concomitant hyperlipidemia^57^).

Our findings have important implications for the management of the atherosclerotic complications of diabetes. Most important, proatherogenic characteristics of myeloid cells and BM precursors persist after normalization of blood glucose. This may help to explain the lack of efficacy of conventional treatments and fundamentally challenges the approach to the management of the vascular risk and complications of diabetes. The identification of metabolic drivers and of particular epigenetic modifications also recommends potential new therapeutic targets.

## Acknowledgments

The authors thank Lisa Heather, Maria da Luz Sousa Fiahlo, and Thomas Milne for their assistance. R.P.C., N.A., and L.E. conceived the study. L.E. performed all in vitro experimentation, gene expression analyses, and immunoblotting described. M.A. contributed to the studies in human samples. C.E.W. and H.G.-A. performed mass spectrometry-based metabolomics. J.T.C. performed human plaque macrophage LCM work and array preparation. L.E. performed Seahorse Bioanalyser experiments under the guidance of J.B. and supervision of M.J.C.. A.L.C. performed cell sorting analysis. L.E. prepared RNA-seq samples. L.E. prepared ATAC-seq samples under T.E.K.’s guidance and I.A.U.’s supervision. N.A. performed all ChIP-sequencing cell preparations. Sequencing was performed by T.K. and A.F.R. under C.B.’s supervision. M.E.L. and A.T.B. ran sequencing data alignment, normalization, and quality control, with L.E. performing downstream analysis. M.E.L. and A.T.B. performed array and sequencing data analysis. K.Z., R.A., and R.C. led animal investigations. L.E., N.A., T.J.C., N.P.R., and M.G.N. participated in experimental design and execution. R.P.C., L.E., N.A., and M.E.L. prepared the manuscript. All authors read and approved the final submission of the manuscript.

## Sources of Funding

This work was supported by the British Heart Foundation Center of Research Excellence Oxford (RE/13/1/30181), British Heart Foundation Project Grant (Drs Akbar and Choudhury: PG/18/53/33895), the National Institute for Health Research Oxford Biomedical Research Center, the Tripartite Immunometabolism Consortium–Novo Nordisk Foundation (grant NNF15CC0018486; Drs Choudhury, Udalova, Rydén, Channon, and Wheelock), Metabolite-Related Inflammation in Diabetes-Spectrum Diseases: New Targets Beyond Glucose (MeRIAD–Novo Nordisk Foundation, grant 0064142; Drs Choudhury, Udalova, Rydén, and Channon), and a 4-year British Heart Foundation PhD studentship and the Doris Field Trust (Dr Edgar). The High-Throughput Genomics Group at the Wellcome Trust Center for Human Genetics was funded by Wellcome Trust grant reference 090532/Z/09/Z and MRC Hub grant G0900747 91070. The Biomedical Sequencing Facility at the CeMM Research Center for Molecular Medicine of the Austrian Academy of Sciences in Vienna Austria. Drs Riksen and Netea received funding from the European Union’s Horizon 2020 research and innovation program under grant agreement 667837. Dr Netea was supported by an ERC Consolidator Grant (No. 310372) and Netherlands Organization for Scientific Research.

## Disclosures

None.

## Supplementary Materials

Data Supplement Figures I–XI

Data Supplement Excel Files I–VII

## Supplementary Material


