# Supplementary material for: Hyperglycemia Induces Trained Immunity in Macrophages and Their Precursors and Promotes Atherosclerosis
Source: Circulation. 2021 Jul 13;144(12):961–82. doi: 10.1161/CIRCULATIONAHA.120.046464 (PMC8448412; doi:10.1161/CIRCULATIONAHA.120.046464)
Supplement: Supplementary file 1 [file cir-144-961-s001.pdf]

Supplemental Material

Hyperglycaemia induces trained immunity in macrophages and their precursors and promotes atherosclerosis

Corresponding Author
Professor Robin P. Choudhury
Division of Cardiovascular Medicine, Radcliffe Department of Medicine, John Radcliffe Hospital, Oxford, OX3 9DU, UK
Telephone +44-1865-234663
Fax +44-1865-234667
E-mail Robin.Choudhury@cardiov.ox.ac.uk

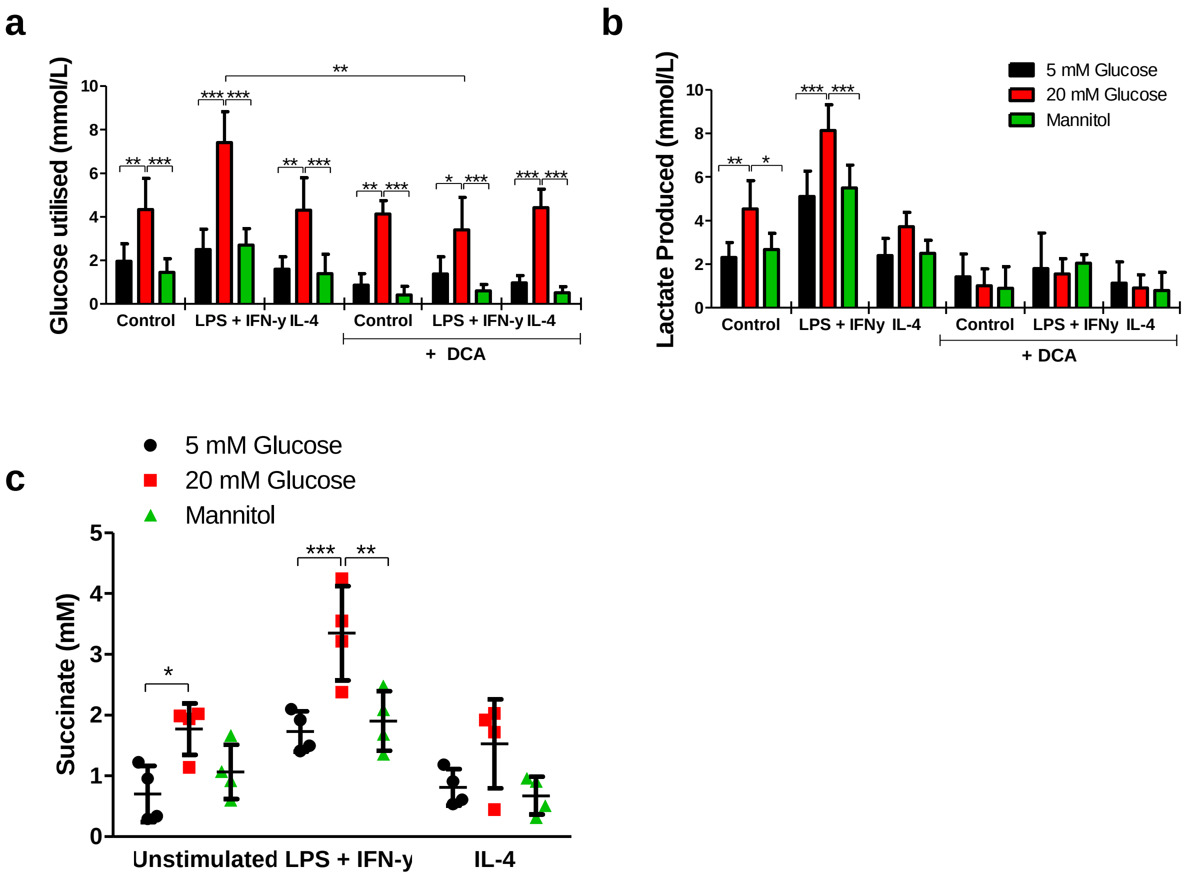

Figure I. High glucose alters BMDM metabolism. Pentra measurements of mouse BMDM culture supernatant displaying (**a**) glucose uptake, (**b**) lactate production in the absence or presence of dichloroacetate (DCA) (n=5) and (**c**) BMDM succinate levels (n=4). All data shown are mean ± SD.; analysed by two-way ANOVA with Bonferroni post-hoc analysis; *p<0.05, **p<0.01, ***p<0.001.

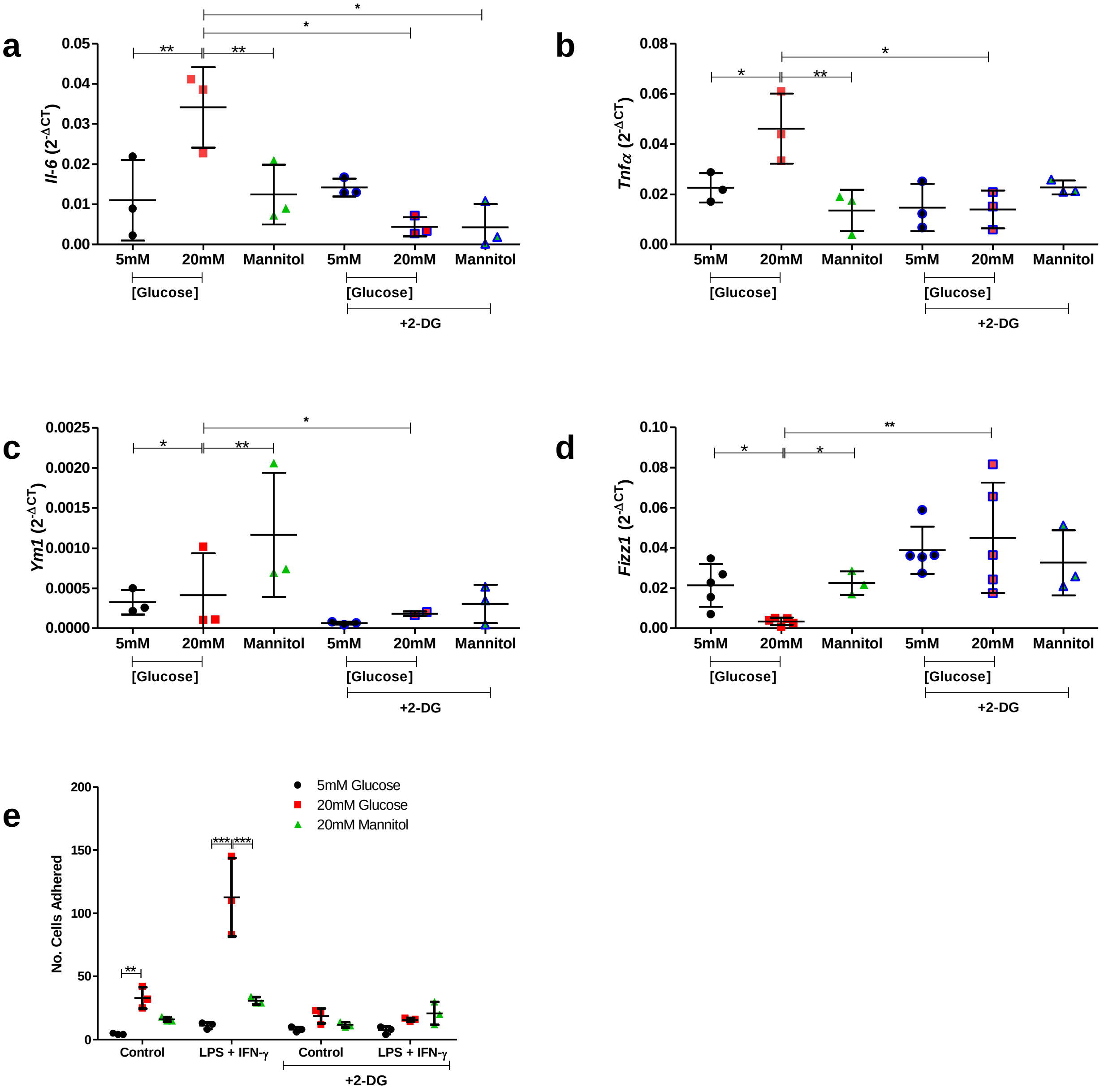

Figure II. 2-deoxy-glucose (2-DG) inhibits BMDM inflammatory gene expression and function. BMDM M1 (LPS + IFN-ɣ stimulated) (**a**) *Il-6* and (**b**) *Tnf-α* gene expression and M2 (IL-4 stimulated) (**c**) *Ym1* and (**d**) *Fizz1* gene expression. qPCR data are normalised to *B2m* expression (n=3). (**e**) Image quantification of BMDM adhesion to endothelial cells (n=3). All data are shown as mean ± SD; (**a-e**) one-way ANOVA or (**e**) two-way ANOVA with Bonferonni Post-Hoc analysis; *p<0.05, **p<0.01, ***p<0.001 and each point represents an individual animal (average 4 images for static adhesion).

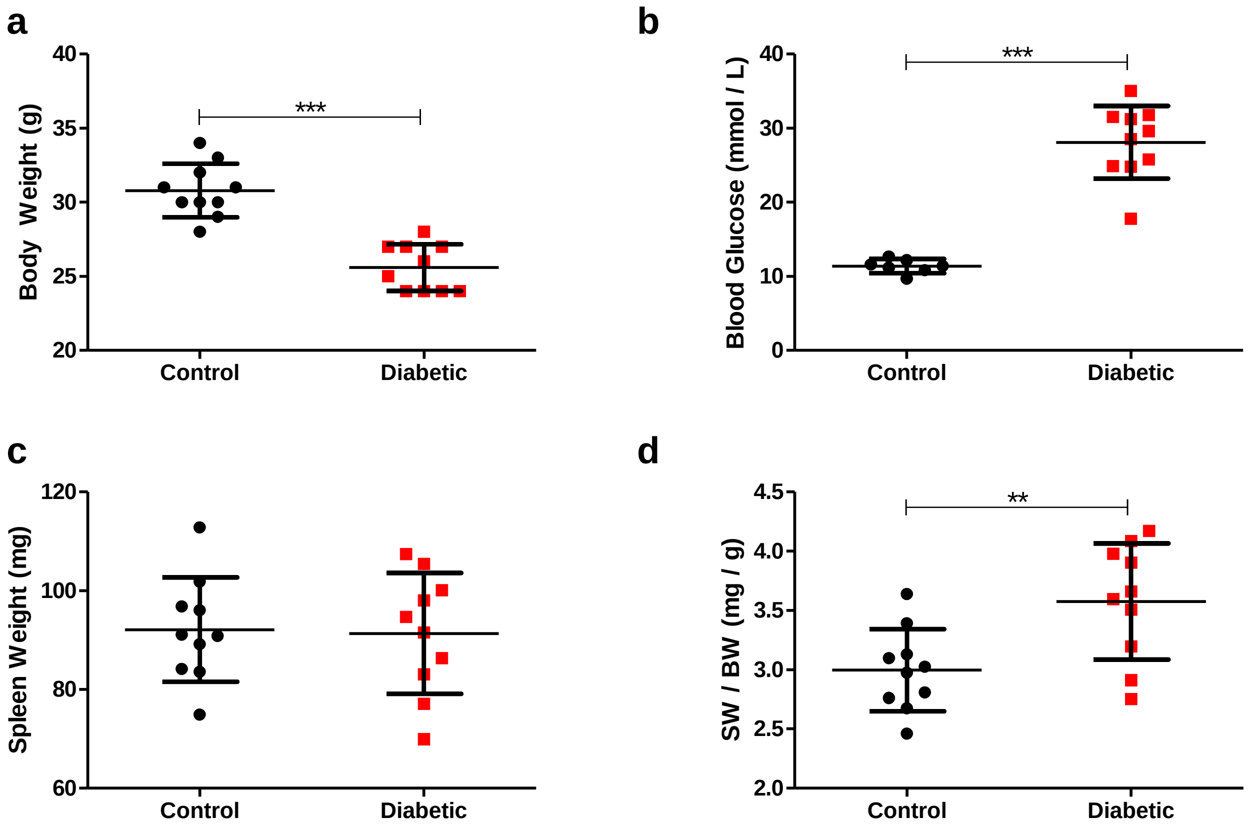

Figure III. Streptozotocin (STZ) diabetic mice characteristics. At time of necropsy, after 6 weeks of maintained in vivo hyperglycaemia, measurements of (**a**) body weight ($p<0.0001$); (**b**) non-fasted blood glucose in millimoles per litre (mmol / L) ($p<0.0001$); (**c**) whole spleen weight in milligram (mg) and (**d**) spleen weight (SW) normalised to body weight (BW) ($p=0.0068$) ($n=10$). Data shown are mean ± SD; analysed by Student's unpaired t-test.

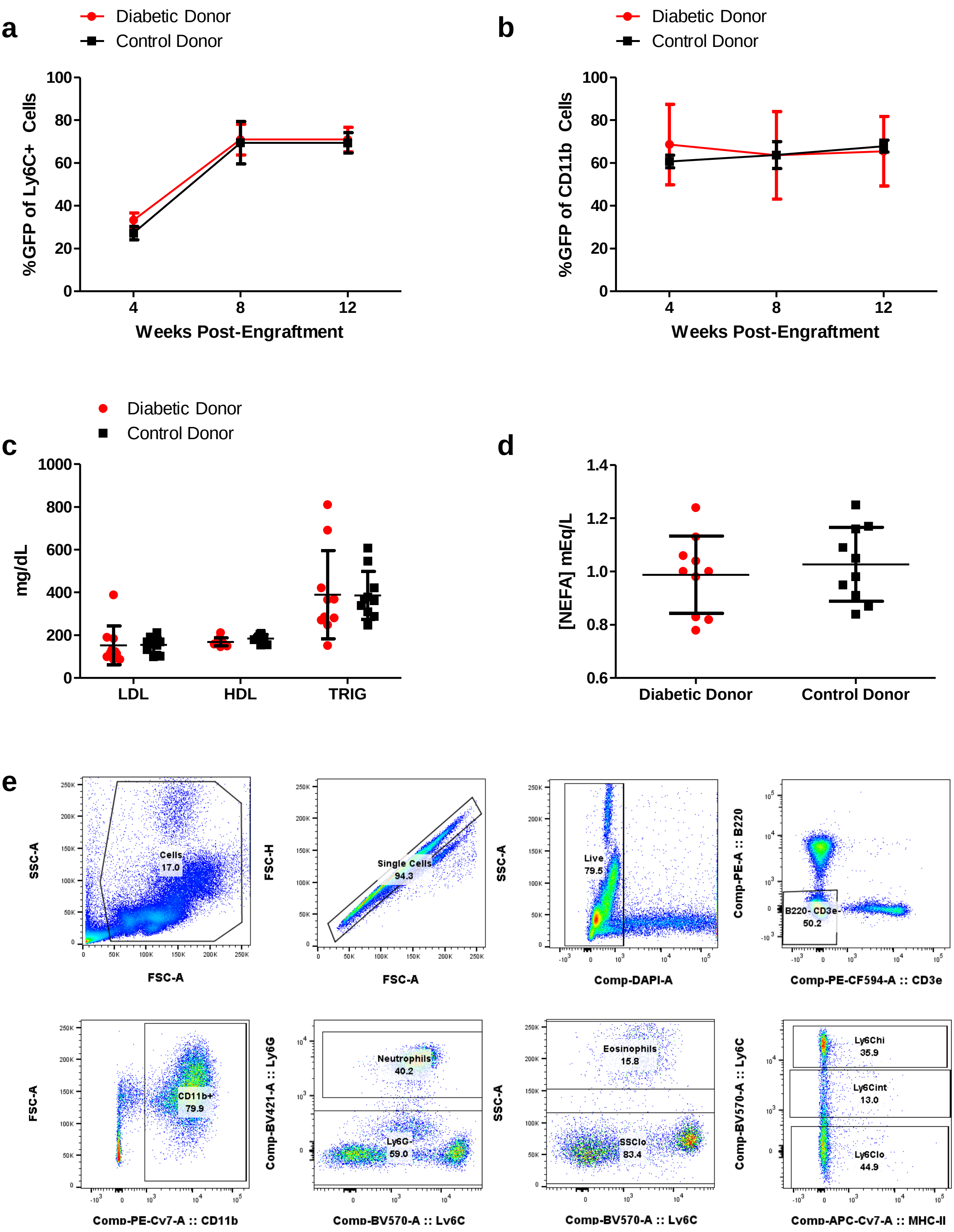

Figure IV. Bone marrow transplant engraftment and lipid measurements. (**a, b**) Flow cytometric analysis of peripheral blood mononuclear cells to measure engraftment by proportion green fluorescent protein (GFP+) cells positive for (**a**) Ly6C or (**b**) CD11b. (C**c**) Serum low density lipoprotein (LDL)-cholesterol, high density lipoprotein (HDL)-cholesterol, triglyceride (TRIG) and (**d**) non-esterified fatty acid (NEFA) measurements post-necropsy. (**e**) Flow cytometry gating strategy for characterising circulating blood leukocyte populations from mice, 12 weeks post engraftment with control or diabetic bone marrow.

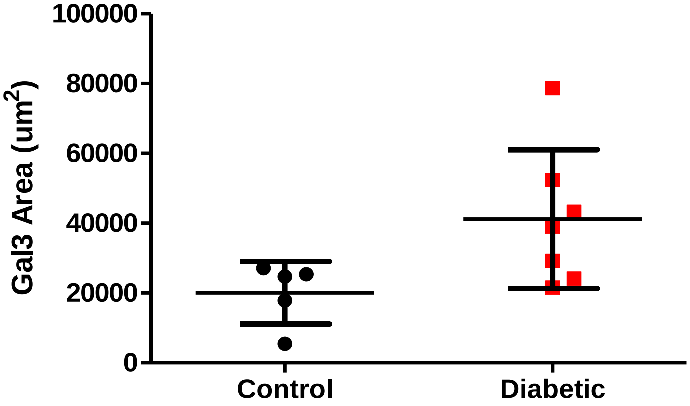

Figure V. Diabetic bone marrow is associated with increased macrophage content, after glucose normalisation. Gal-3 immunostaining (Data shown as mean ± SD, analysed by t-test, control n=5, diabetic n=7; P = 0.052)

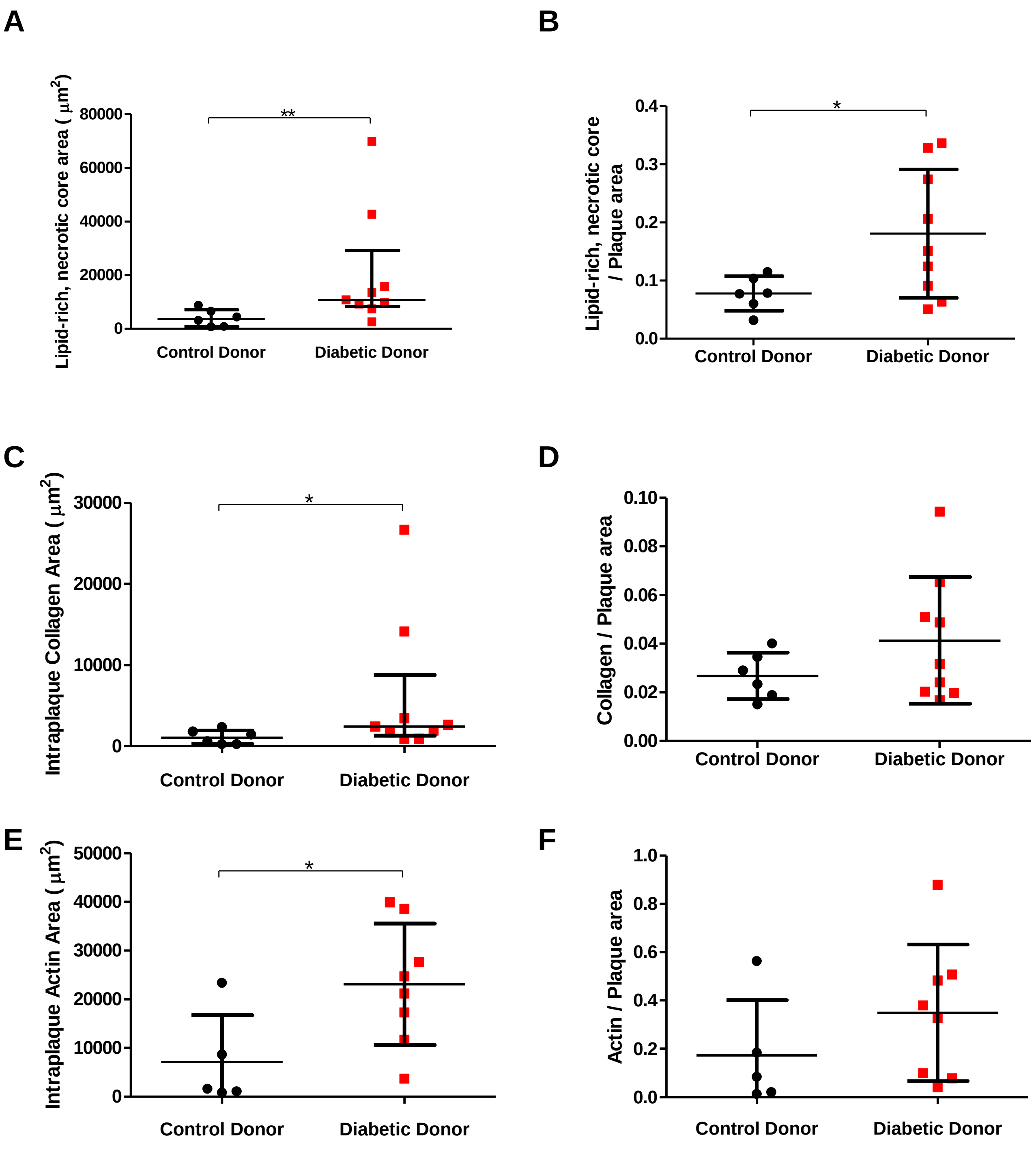

Figure VI. Diabetic bone marrow drives larger lipid-rich necrotic core in plaques, after glucose normalisation. Masson Trichrome image quantification of lipid-rich necrotic core regions (**a**) before ($p=0.0076$) and (**b**) after normalisation to plaque area ($p=0.046$), and quantification of intra-plaque collagen (**c**) before ($p=0.036$) or (**d**) after normalisation to plaque area. Immunofluorescence image quantification of smooth muscle cell content (α-actin stained) (**e**) before and (**f**) after normalisation to plaque area. Data shown as (**a, c**) median ± interquartile range, analysed by Mann-Whitney U test or (**b, d, e & f**) mean ± SD and analysed by unpaired t-test; n=5-10.

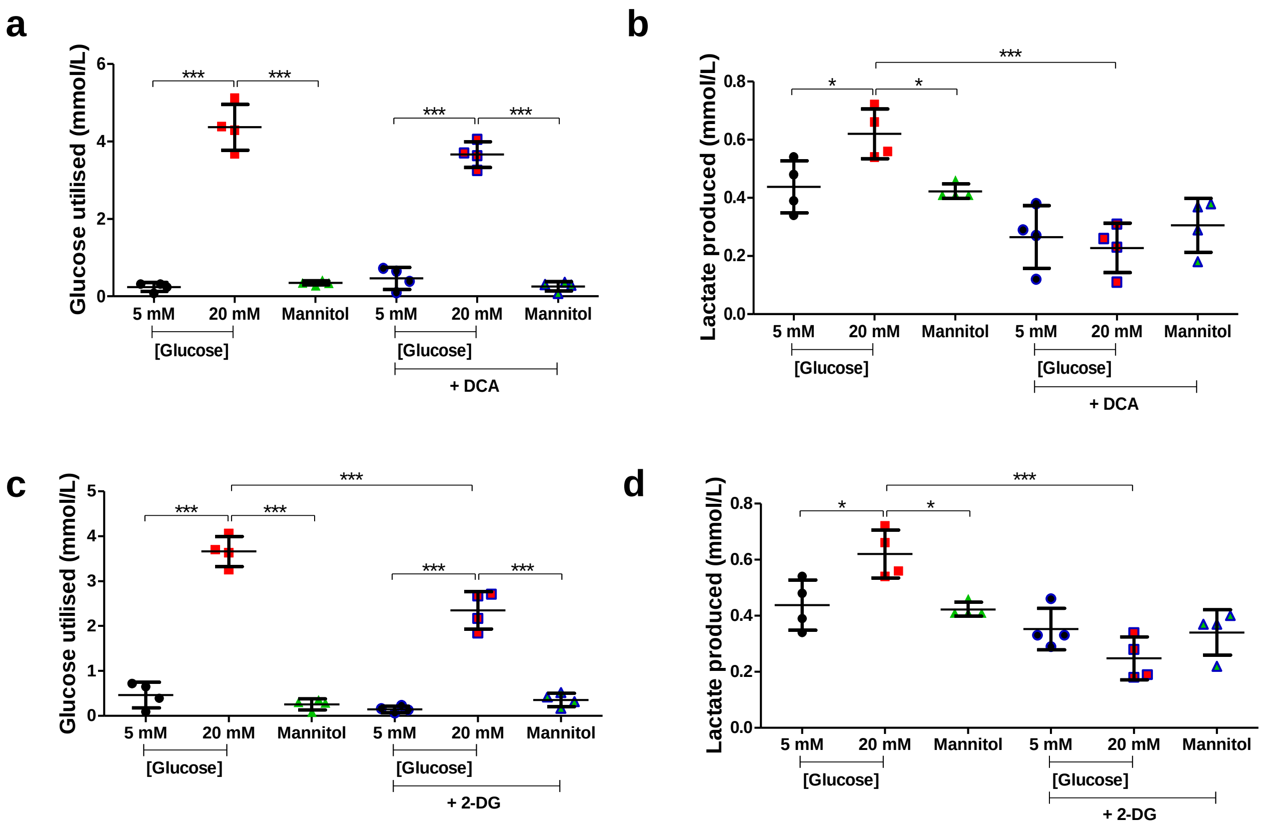

Figure VII. High glucose alters haematopoietic stem cell (HSC) metabolism. Pentra measurements of mouse HSC culture supernatant displaying (**a**) glucose uptake and (**b**) lactate production in the absence or presence of dichloroacetate (DCA) or (**c**) glucose uptake and (**d**) lactate production in the absence or presence of 2-deoxyglucose (2-DG) (n=4). All data shown are mean ± SD.; analysed by two-way ANOVA with Bonferroni post-hoc analysis; *p<0.05, ***p<0.001.

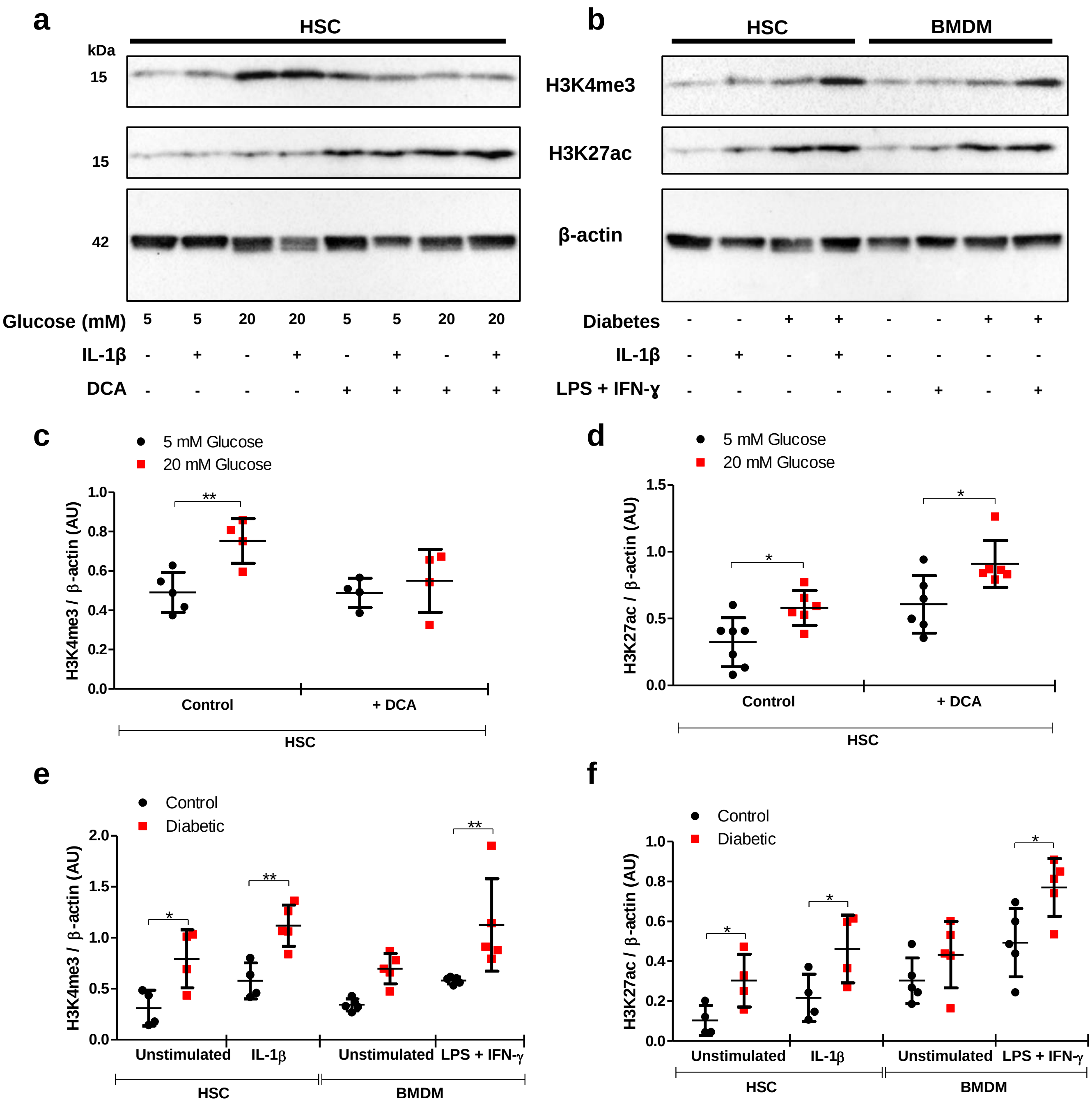

Figure VIII. Epigenetic changes induced by high glucose or diabetes. Example western blots with (**a**) haematopoietic stem cells (HSC) in varying glycaemic conditions ± dichloroacetate (DCA) and (**b**) HSC or BMDM derived from control or diabetic mice, in unstimulated or pro-inflammatory stimulated conditions. Quantification of western blot band intensity for (**c**) H3K4me3 or (**d**) H3K27ac (normalised to loading control β-actin) in control HSC in varying glycaemic conditions or for (**e**) H3K4me3 or (**f**) H3K27ac (normalised to loading control β-actin) for control or diabetic derived HSC or BMDM (average 2 blots per data point; n=5; samples derive from the same experiment and blots were processed in parallel).Each data point represents an individual animal (average 6 sections); n= 7-10. All data are shown as mean ± SD; (**c**-**f**) two-way or (**h**) one-way ANOVA analysis; *p<0.05, **p<0.01.

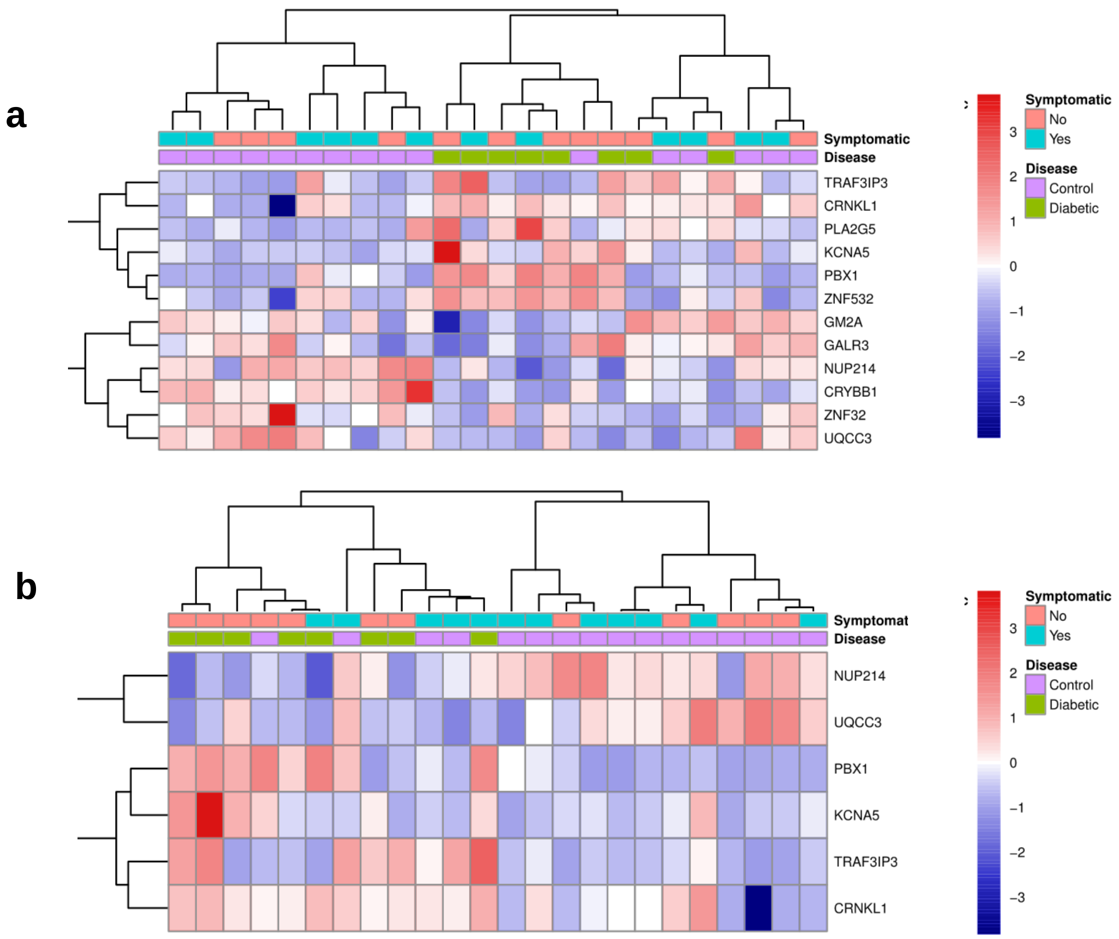

Figure IX. RUNX1 target genes discovered among genes differentially expressed (DE) from diabetic or non-diabetic control laser capture microdissected human plaque macrophages, as discovered at p-value (**a**) $p<0.05$ or (**b**) $p<0.01$. Row-normalized log2 values are shown for genes differentially expressed in diabetic (green, n=8) and control (purple, n=16) samples.

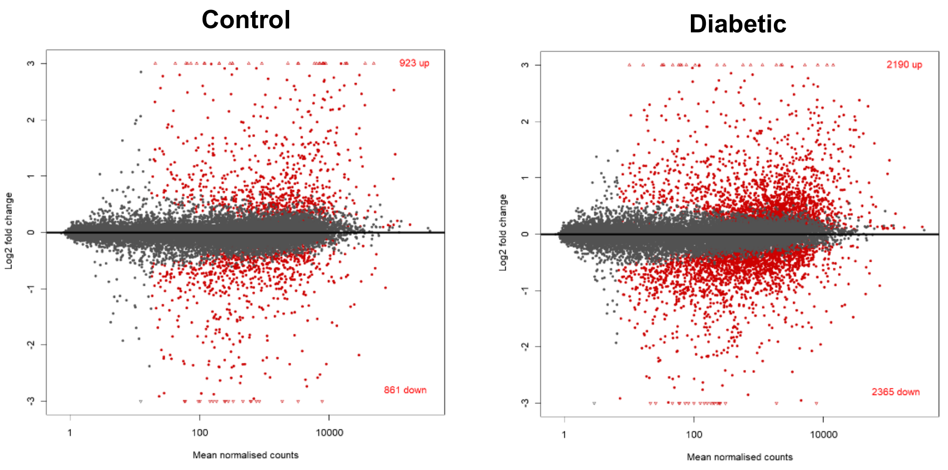

Figure X. MA plots of mRNA changes after M1 stimulation of human PBMC from control and people with diabetes. Differential gene expression determined by DESeq2, red highlighted points adjusted p-value < 0.05, control n=4, diabetic n=6.

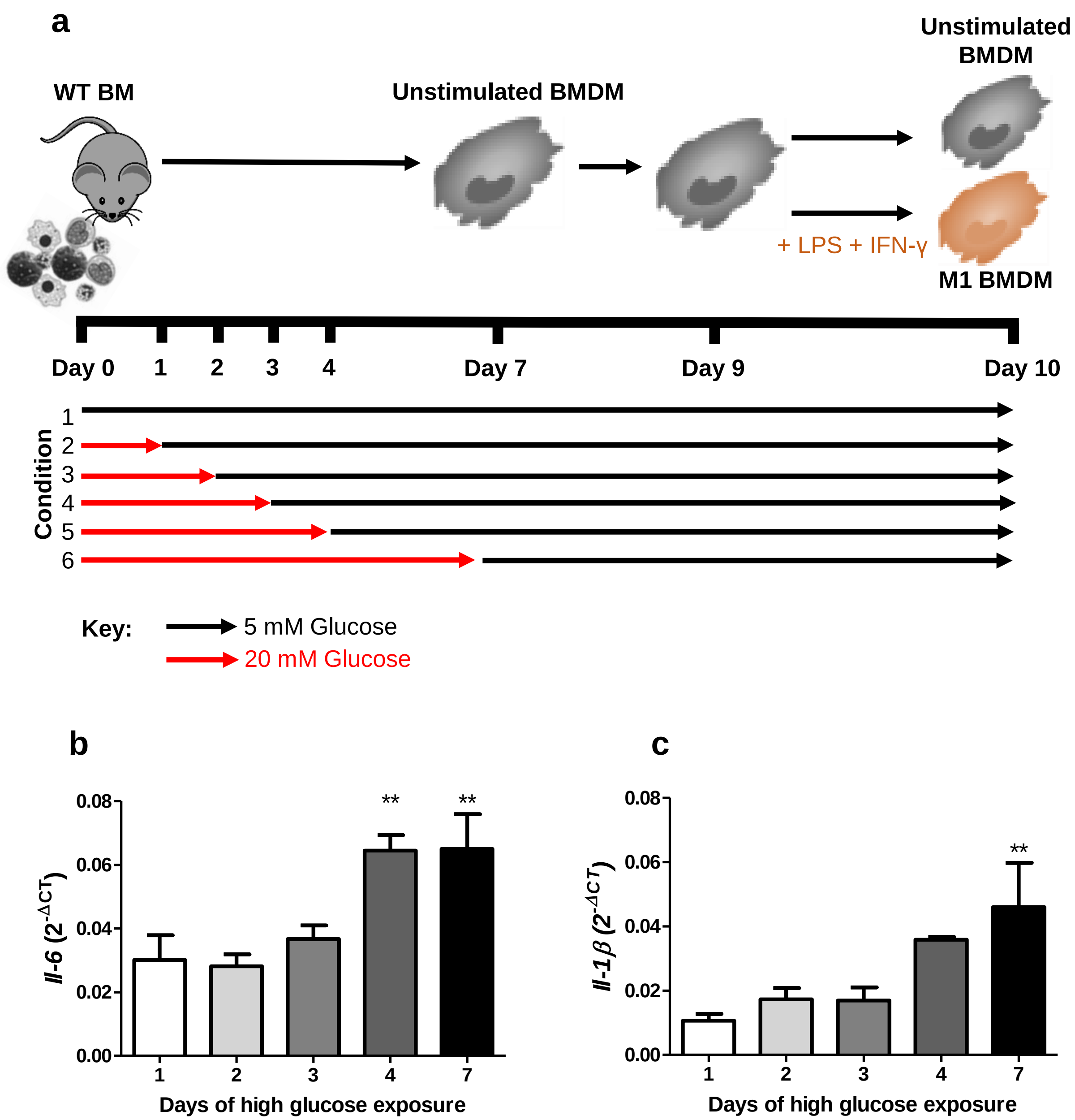

Figure XI. Hyperglycaemic memory can be induced in BMDM in vitro in 4 days. Wild-type (WT) bone marrow (BM) was differentiated into bone marrow derived macrophages (BMDM) for varying periods of physiological (5 mM) or high glucose (20 mM), (**a**) as illustrated. After LPS + IFNγ stimulation, M1 BMDM (**b**) *Il-6* and (**c**) *Il-1β* were assessed by qPCR; normalised to B2m gene expression. Sample n=3-5. Data displayed as mean ± SD, 1-way ANOVA with Bonferroni Post-Hoc, **p<0.01 (individual comparison between day 4 or 7 vs day 1, 2 or 3 data).

Supplementary Excel Tables Legends

I. **HSC ATAC-seq peak processing.** Summary of sample conditions used for HSC ATAC-seq experimentation and of the read number during peak processing. ChrM = chromosome M.

II. **Differential HSC ATAC-seq peak analysis.** Table highlights the comparisons made, each sample's corresponding FRiP score (fraction of reads in called peak) and a summary of the number of differential peaks identified.

III. **Unstimulated BMDM differentially expressed (DE) genes.** Full list of DE genes (adjusted p-value <0.05 and fold-change >1.5) identified in unstimulated control and diabetic BMDM RNA-seq analysis.

IV. **LPS + IFNɣ BMDM stimulated differentially expressed (DE) genes.** Full list of DE genes (adjusted p-value <0.05 and fold-change >1.5) identified in LPS + IFNɣ control and diabetic BMDM RNA-seq analysis.

V. **RUNX1 target genes are over represented in diabetic LPS + IFNɣ BMDM.** Full list of (DE) genes (adjusted p-value <0.05 and fold-change >1.5) in LPS + IFNy stimulated BMDM, as assessed by RNA-seq, with RUNX1 target genes marked.

VI. **Patient characteristics.** Summary of the carotid endarterectomy patients whose plaque macrophages were used for LCM isolation and array analysis, including body mass index (BMI), glycated haemoglobin (HbA1c)

VII. **BMDM ATAC-seq normalised counts.** All BMDM ATAC-seq log-normalised counts used to generate heat maps and other plots.
